# Multiset sparse partial least squares path modeling for high dimensional omics data analysis

**DOI:** 10.1186/s12859-019-3286-3

**Published:** 2020-01-09

**Authors:** Attila Csala, Aeilko H. Zwinderman, Michel H. Hof

**Affiliations:** Department of Clinical Epidemiology, Biostatistics and Bioinformatics, University of Amsterdam, Amsterdam, 1105 AZ The Netherlands

**Keywords:** Multivariate analysis, High dimensional omics data, Partial least squares

## Abstract

**Background:**

Recent technological developments have enabled the measurement of a plethora of biomolecular data from various omics domains, and research is ongoing on statistical methods to leverage these omics data to better model and understand biological pathways and genetic architectures of complex phenotypes. Current reviews report that the simultaneous analysis of multiple (i.e. three or more) high dimensional omics data sources is still challenging and suitable statistical methods are unavailable. Often mentioned challenges are the lack of accounting for the hierarchical structure between omics domains and the difficulty of interpretation of genomewide results. This study is motivated to address these challenges. We propose multiset sparse Partial Least Squares path modeling (msPLS), a generalized penalized form of Partial Least Squares path modeling, for the simultaneous modeling of biological pathways across multiple omics domains. msPLS simultaneously models the effect of multiple molecular markers, from multiple omics domains, on the variation of multiple phenotypic variables, while accounting for the relationships between data sources, and provides sparse results. The sparsity in the model helps to provide interpretable results from analyses of hundreds of thousands of biomolecular variables.

**Results:**

With simulation studies, we quantified the ability of msPLS to discover associated variables among high dimensional data sources. Furthermore, we analysed high dimensional omics datasets to explore biological pathways associated with Marfan syndrome and with Chronic Lymphocytic Leukaemia. Additionally, we compared the results of msPLS to the results of Multi-Omics Factor Analysis (MOFA), which is an alternative method to analyse this type of data.

**Conclusions:**

msPLS is an multiset multivariate method for the integrative analysis of multiple high dimensional omics data sources. It accounts for the relationship between multiple high dimensional data sources while it provides interpretable results through its sparse solutions. The biomarkers found by msPLS in the omics datasets can be interpreted in terms of biological pathways associated with the pathophysiology of Marfan syndrome and of Chronic Lymphocytic Leukaemia. Additionally, msPLS outperforms MOFA in terms of variation explained in the chronic lymphocytic leukaemia dataset while it identifies the two most important clinical markers for Chronic Lymphocytic Leukaemia

**Availability:**

http://uva.csala.me/mspls.https://github.com/acsala/2018_msPLS

## Background

Technological developments have enabled the measurement and storage of a plethora of biomolecular data extracted from various omics domains, such as data from the genome, epigenome, proteome or metabolome. It has become common to measure hundreds of thousands of biomolecular variables. To explore biological pathways across multiple omics domains, which might be associated with phenotypic (e.g. disease) outcomes, a natural research direction is to simultaneously analyse these omics domains. Complex diseases, such as obesity, diabetes, and schizophrenia have genetic architectures that involve many biological pathways, since they are a result of interactions between genomic, epigenomic and environmental variables [1, 2]. Therefore, modeling biological pathways across multiple omics domains might help to better understand the underlying genetic architecture and biological processes of complex phenotypes, which in turn leads to improved diagnosis, prognosis and therapy [1].

There is ongoing research for suitable statistical methods that could help leverage the available omics data to better model and understand biological pathways and genetic architectures of complex phenotypes on the biomolecular level [3].

Some of the first statistical methods developed for the integrated (i.e. simultaneous) analysis of multiple high dimensional omics datasets are generalizations of well known multivariate methods; e.g. sparse Canonical Correlation Analysis (CCA) [4–8], sparse Redundancy Analysis (RDA) [9, 10], and Multi-Omics Factor Analysis (MOFA) [11]. Detailed reviews and discussions on multivariate methods for omics data analysis can be found in [3, 12–18]. Although there are various statistical methods available to analyse omics data, recent reports argue that the simultaneous analysis of multiple (i.e. three or more) omics data sources is still challenging and current statistical methods are suboptimal. Among the challenges are the lack of accounting for the hierarchical structure between omics domains (i.e. relationship between data sources) and the difficulty of interpretation of genomewide results [2, 3, 19, 20].

To address those challenges, we propose a multiset multivariate statistical method, called multiset sparse Partial Least Squares path modeling (msPLS). msPLS is the penalised extension of multi-block Partial Least Squares path modeling (PLS-PM). Given the situation where biomolecular variables from multiple omics domains are measured on the same patients with shared phenotypes of interest, msPLS models biological pathways by identifying biomarkers (i.e. biomolecular variables that are associated with the phenotypes of interest) in each omics domain. The omics domains are assumed to have a hierarchical structure between each other, and their relationship is modelled in terms of dependencies through explanatory and response domain pairs. The explanatory and response omics data source pairs can be determined through the hypothesised information transfer between data sources as follows [21]. In an asymmetric relationship, a response data source is dependent on a explanatory data source if the prevalent way of information transfer is from the explanatory to the response data source. In a symmetric relationship, there is a recursive information transfer between data sources, and both data sources are dependent on each other. In PLS-PM, latent variables (LVs) are used to model the relationships between explanatory and response manifest variables (MVs) [22, 23]. Similarly to PLS-PM, the LVs in msPLS are linear combinations of the MVs, and are estimated in an iterative regression framework [24]. The LVs are constructed so that the combination of the explanatory MVs account for the most variance either directly in the response MVs (in an asymmetric relationship), or in the LVs of the response MVs (in a symmetric relationship). In general, Partial Least Squares path modeling distinguishes between these two types of relationships between data sources (i.e. symmetric or asymmetric relationships) the same way as the two well known multivariate statistical methods Canonical Correlation Analysis [8] and Redundancy Analysis [10] do. In the “Methods” section, we describe msPLS’s direct correspondence with those two well known multivariate methods. We give a detailed description of msPLS in the “Methods” section.

To illustrate such an explanatory and response dependency structure, consider that we have biomolecular variables (i.e. genomewide epigenomic, transcriptomic and proteomic variables) measured in patients with Marfan syndrome. The goal of this analysis is to use msPLS to explore biological pathways associated with Marfan syndrome, through the simultaneous analysis of the data sources. For this setting, we assume that the proteomic variables are responses for both the epigenomic and transcriptomic variables. Thus the proteome data source has an asymmetric relationship with both the epigenome and the transcriptome data sources. Additionally, there is a symmetric relationship between the epigenome and the transcriptome data sources, assuming a recursive information transfer between the epigenome and transcriptome. These assumptions are based on the special biological sequential information transfers of the central dogma of molecular biology and its elaborated versions [25, 26]. Given the above relationship between omics domains, msPLS identifies the combination of epigenomic and transcriptomic biomarkers that explain the most variance in the proteomic variables, while the combination of the epigenomic and transcriptomic biomarkers have maximum possible correlation with each other. This example is elaborated in more detail in the “Results” section of this paper.

To provide interpretable results from analyses of hundreds of thousands of MVs is addressed through sparse variable selection. msPLS enforces sparse variable selection through penalization methods, such as through the Least Absolute Shrinkage and Selection Operator (LASSO), Ridge, and Elastic Net (ENet) penalization methods [27]. These penalization methods are introduced to PLS-PM by regularising the multivariate regression steps in the iterative regression framework. Introducing regularisation allows msPLS to deal with the characteristic high dimensionality of omics datasets, where the number of variables are much higher than the number of samples. In addition, regularisation improves the interpretability of the final model in the form of sparse variable selection. Once the final model is obtained, the identified biomarkers can be interpreted in terms of biological pathways that are associated with the interest of phenotypes. In the “Methods” section, we quantify msPLS’s ability to identify a handful of associated variables from multiple data sources among thousands of irrelevant variables.

The rest of the paper is structured as follows. In the next section, the results of the real data analyses are described, where msPLS was applied to geneomewide biomolecular variables measured in Marfan patients in order to explain the variance in the phenotypic proteomic variables with the combination of biomarkers from the epigenome and transcriptome, while accounting for a hypothesised relationship in omics domains. Additionally, msPLS was applied to a second omics dataset containing data from patients with Chronic lymphocytic leukaemia, and its results were compared to the results of MOFA. We discuss these findings in the “Discussion” section. In the “Methods” section, we describe msPLS and its implementation in an iterative regression framework, along with a working example of the analysis of three related data sources. In addition, we describe how msPLS, and PLS in general, relate to two well known multivariate methods, CCA and RDA. Finally, we show the results from a simulation study that was performed to assess the ability of msPLS to deal with high dimensional data and its ability to extract explanatory MVs that explain the most variance in the response MVs and LVs.

## Results

We applied msPLS to genomewide epigenomic, transcriptomic and proteomic data sources measured in Marfan patients [28]. In addition, we applied msPLS to genomic, epigenomic, transcriptomic, and drug response data sources measured in Chronic Lymphocytic Leukaemia (CLL) patients [29].

### Marfan data

The goal of this analysis was to explore biological pathways associated with Marfan disease based on epigenomic, transcriptomic and proteomic data measured in 37 Marfan patients [30]. The 364,134 epigenomic methylation variables were obtained by Illumina Infinium HumanMethylation450 BeadChip from blood leukocytes, the 18,424 transcriptomic gene expression variables were obtained by Affymetrix Human Exon 1.0ST Arrays from skin biopsy, and the 47 proteomic cytokine variables were measured in blood plasma.

**Fig. 1:**
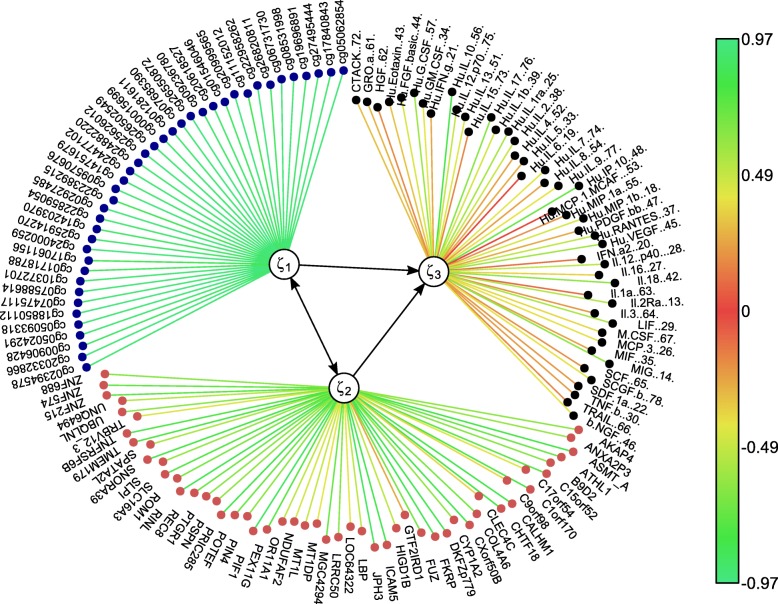
msPLS identified a combination of 40 epigenomic markers (denoted as ***ξ***_1_) and 52 transcriptomic markers (denoted as ***ξ***_2_) that explain the most variance in the proteome variables. The color scale represents the strength of **w** regression weights

**Fig. 2:**
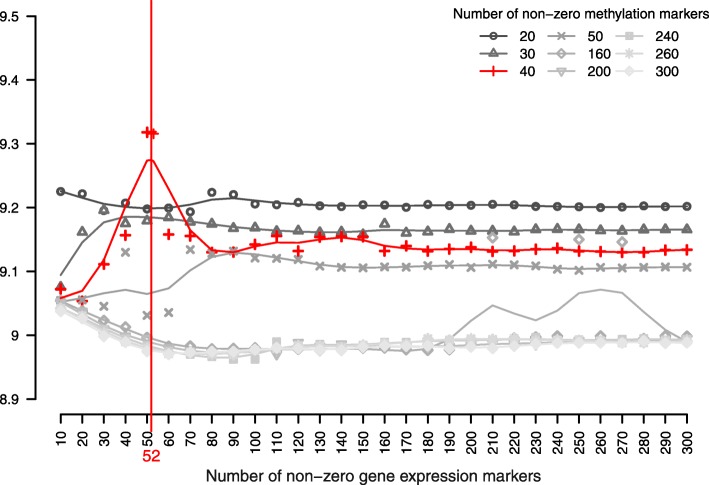
msPLS identified 40 methylation markers and 52 gene expression markers that optimised the sum of squared correlation of the explanatory LVs of the epigenome and transcriptome with the MVs from the proteome

The model was constructed by extracting the combination of LVs from the epigenome and transcriptome that explain the most variance in the phenotypic proteome MVs (Fig. 1). We hypothesised a symmetric relationship between the epigenome and transcriptome and asymmetric relationships from the proteome to both the epigenome and the transcriptome, so that the proteomic variables were set as response MVs for both the epigenomic and transcriptomic MVs. We used Univariate Soft Thresholding (UST) penalisation with 10-fold cross validation (see “Methods” section) to find the penalisation parameter that optimised the sum of squared correlations between the combination of LVs from the epigenome and transcriptome with respect to the proteome variables (see Eq. (5) in Methods). The final model extracted 40 methylation markers and 52 gene expression markers that optimised the sum of squared correlation of the explanatory LVs of the epigenome and transcriptome with the MVs from the proteome (Fig. 2). The sum of squared correlations was 9.32. Through bootstrapping, we obtained a 95% confidence interval of [9.03, 9.56] and a *p*-value <0.01 after permutation (see “Methods” section). The best fitting model resulted in a set of LVs that captured 49% of variance in both the epigenome and transcriptome variables and 65% of variance in the proteome variables. The extracted biomarkers with their corresponding individual contribution towards the overall explained variance in the proteomic variables (i.e. illustrated by the methylation and gene expression weights) and the proteomic variables with their corresponding individual correlation strength with the combination of the explanatory LVs (i.e. illustrated by the cytokine weights) are listed in Table 1.

**Table 1:** The weights of the epigenomic, transcriptomic and proteomic variables extracted by msPLS from the Marfan data

Methylation markers		Gene expression markers		Cytokine markers	
Site	**w**	Gene code	**w**	Marker code	**w**
cg02394578	0.93	AKAP4	0.65	b NGF 46	0.43
cg20332866	0.96	ANXA2P3	0.63	CTACK 72	0.34
cg00906428	0.91	ASMT_A	0.83	GRO a 61	0.31
cg05024291	0.93	ATHL1	0.73	HGF 62	0.21
cg05093318	0.95	B9D2	0.66	Hu Eotaxin 43	-0.34
cg18850112	0.97	C15orf52	0.76	Hu FGF basic 44	0.46
cg07475117	0.95	C17orf54	0.44	Hu G CSF 57	0.61
cg07588614	0.94	C1orf170	0.8	Hu GM CSF 34	0.43
cg10372701	0.94	C9orf98	0.4	Hu IFN g 21	0.28
cg01718788	-0.9	CALHM1	0.84	Hu IL 10. 56	0.82
cg17061156	0.87	CHTF18	0.81	Hu IL 12 p70. 75	0.51
cg24000259	0.95	CLEC4C	0.39	Hu IL 13 51	0.44
cg25914270	0.92	COL4A6	0.69	Hu IL 15 73	0.19
cg14203970	0.91	CXorf50B	0.84	Hu IL 17 76	0.65
cg22859054	0.94	CYP1A2	0.64	Hu IL 1b 39	0.48
cg02927485	0.96	DKFZp779	0.85	Hu IL 1ra 25	0.58
cg22389215	0.88	FKRP	0.85	Hu IL 2 38	0.49
cg09570676	0.96	FUZ	0.71	Hu IL 4 52	0.26
cg14751679	0.93	GTF2IRD1	0.29	Hu IL 5 33	0.29
cg24477102	0.94	HIGD1B	0.53	Hu IL 6 19	0.04
cg24882220	0.93	ICAM5	0.85	Hu IL 7 74	0.44
cg25626012	0.95	JPH3	0.88	Hu IL 8 54	-0.26
cg26502549	0.94	LBP	0.53	Hu IL 9 77	0.42
cg00015699	0.93	LOC64322	-0.44	Hu IP 10. 48	0.72
cg01281611	0.93	LRRC50	0.66	Hu MCP 1	-0.04
cg07685390	0.95	MGC4294	0.73	Hu MIP 1a 55	0.23
cg26550872	0.97	MT1DP	0.43	Hu MIP 1b 18	0.47
cg09236780	0.92	MT1L	0.51	Hu PDGF bb 47	-0.29
cg20618527	0.92	NDUFAF2	-0.47	Hu RANTES 37	0.52
cg01546046	0.89	OR11A1	-0.53	Hu VEGF 45	0.59
cg20999565	0.96	PEX11G	0.77	IFN a2 20	0.15
cg11152012	0.87	PIF1	0.88	Il 12 p40 28	0.52
cg22958262	0.96	PIN4	0.65	Il 16 27	0.38
cg26820811	0.9	POTEF	-0.72	Il 18 42	0.64
cg06731730	0.95	PRIC285	0.83	Il 1a 63	0.14
cg08531998	0.94	PSPN	0.63	Il 2Ra 13	0.56
cg19696891	0.92	PTGR1	-0.65	Il 3 64	0.24
cg27495444	0.93	REC8	0.72	LIF 29	0.57
cg17840843	0.93	RINL	0.85	M CSF 67	0.4
cg05062854	0.95	ROM1	0.69	MCP 3 26	0.42
		SLC16A3	0.78	MIF 35	0.27
		SLPI	-0.63	MIG 14	0.63
		SNORA39	0.81	SCF 65	0.23
		SPATA2L	0.57	SCGF b 78	0.42
		TMEM179	0.79	SDF 1a 22	0.24
		TNFRSF6B	0.79	TNF b 30	0.26
		TRBV12_3	0.46	TRAIL 66	-0.2
		UBQLNL	0.65		
		UNQ6494	0.55		
		ZNF215	-0.76		
		ZNF574	0.74		
		ZNF688	0.65		

**Fig. 3:**
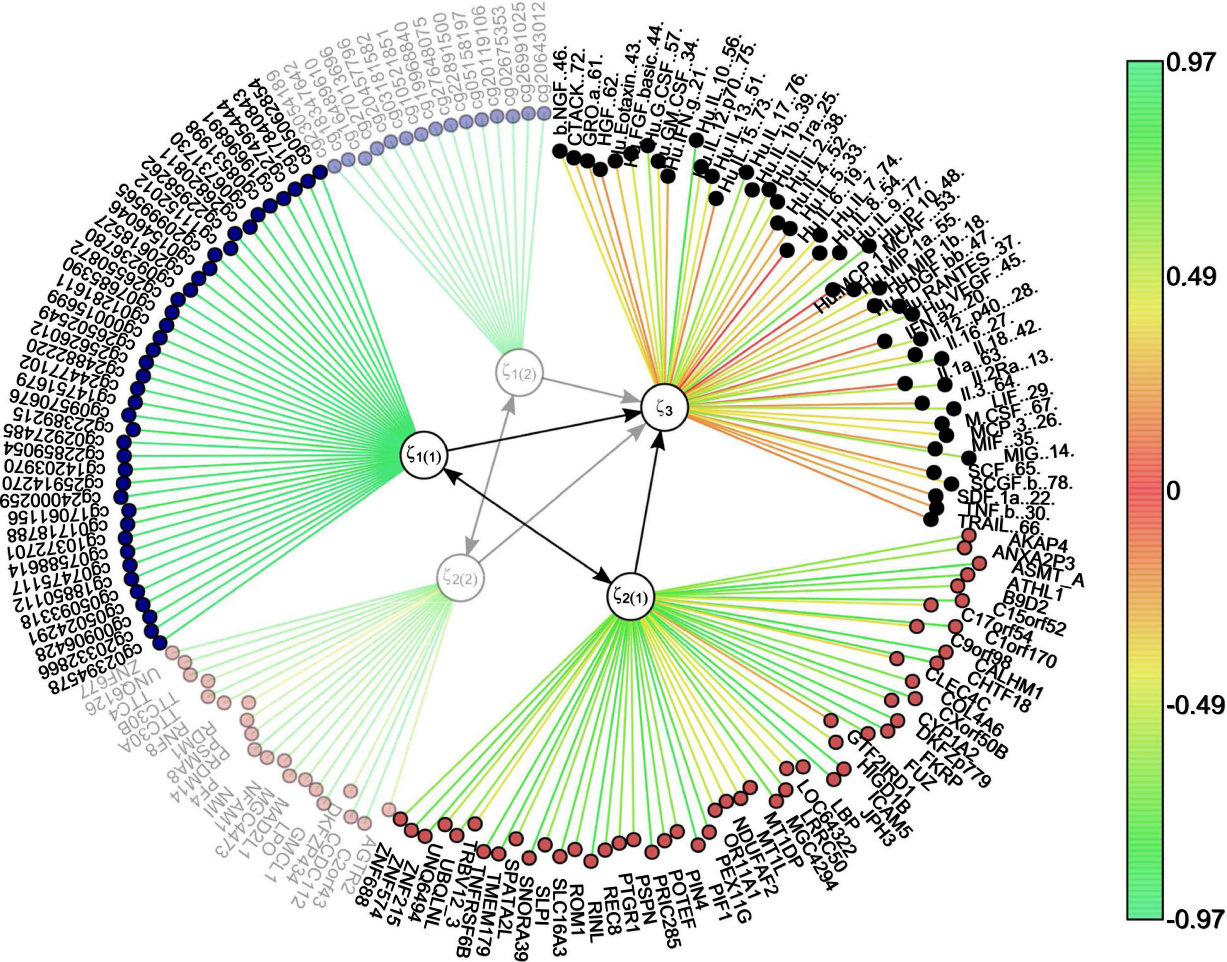
The resulting model from Section 3.2 extended to two LVs per dataset. The first set of LVs ***ξ***_1(1)_ and ***ξ***_2(1)_ partition out a different portion of variance in the proteome MVs than the second set of LVs ***ξ***_1(2)_ and ***ξ***_2(2)_. The colour scale represents the strength of **w** regression weights

Subsequent set of LVs can be extracted by applying msPLS to the residual data of the epigenome and transcriptome data sources and to the original proteome data source (see “Methods” section). Doing so, we obtained a second set of LVs that explain a different portion of variance in the MVs than the first set of LVs (Fig. 3). After optimizing the model on the residual data, we obtained the second set of LVs that captured 67% of the remaining variance in both the epigenome and transcriptome variables and 91% of the remaining variance in the proteome variables. Thus the first two sets of LVs captured a total of 83% variance in the epigenome and transcriptome variables and a total of 97% variance in the proteome variables. The list of the second set of epigenomic and transcriptomic biomarkers and the proteomic variables with their corresponding weights can be found in Table 2.

**Table 2:** The second set of weights of the epigenomic, transcriptomic and proteomic variables extracted by msPLS from the Marfan data

Methylation markers		Gene expression markers		Cytokine markers	
Site	**w**	Gene code	**w**	Marker code	**w**
cg23054189	0.93	AGTR2	-0.59	b NGF 46	0.57
cg18347642	0.93	C2orf43	0.87	CTACK 72	0.52
cg16489610	0.85	CCDC112	0.87	GRO a 61	0.16
cg27013696	0.91	DKFZP434	-0.58	HGF 62	-0.08
cg20457796	0.93	GMCL1	0.88	Hu Eotaxin 43	0.12
cg03181582	0.91	LPO	-0.77	Hu FGF basic 44	0.23
cg10521851	0.9	MAD2L1	0.74	Hu G.CSF 57	-0.57
cg19968840	0.92	MGC4473	-0.81	Hu GM CSF 34	-0.03
cg27648075	0.92	NFAM1	-0.7	Hu IFN g 21	-0.03
cg22891500	0.92	NMI	0.8	Hu Il 10 56	0.17
cg05158197	0.92	PF4	-0.83	Hu IL 12 p70 75	0.43
cg20119106	0.93	PRDM14	-0.71	Hu IL 13 51	0.26
cg02675353	0.91	PSMA8	-0.63	Hu IL 15 73	0.67
cg26991025	0.93	RDM1	0.47	Hu IL 17 76	-0.14
cg20643012	0.92	RNF8	0.69	Hu IL 1b 39	-0.03
		TTC30A	0.8	Hu IL 1ra 25	-0.03
		TTC30B	0.68	Hu IL 2 38	0.36
		TTC4	0.81	Hu IL 4 52	0.67
		UNQ6126	-0.79	Hu IL 5 33	0.23
		ZNF677	0.86	Hu IL 6 19	-0.04
				Hu IL 7 74	0.44
				Hu IL 8 54	-0.69
				Hu IL 9 77	0.13
				Hu IP 10 48	0.73
				Hu MCP 1 MCAF 53	-0.09
				Hu MIP 1a 55	-0.24
				Hu MIP 1b 18.	0.26
				Hu PDGF bb 47	0.3
				Hu RANTES 37	0.84
				Hu VEGF 45	0.69
				IFN a2 20	-0.29
				Il 12 p40 28	0.59
				Il 16 27	0.35
				Il 18 42	0.4
				Il 1a 63	0.04
				Il 2Ra 13	0.57
				Il 3 64	0.18
				LIF 29	-0.04
				M CSF 67	0.24
				MCP 3 26	0.19
				MIF 35	-0.48
				MIG 14	0.24
				SCF 65	-0.11
				SCGF b 78	0.65
				SDF 1a 22	0.39
				TNF b 30	-0.19
				TRAIL 66	0.38

A gene set enrichment analysis (available at https://reactome.org) was used to test the association of the resulting gene expression markers (see Table 1) with already known biological pathways. The gene set enrichment analysis identified 208 pathways (see Additional file 2). We ordered the pathways on their respective *p*-values from an over-representation analysis (see https://reactome.org). For the sake of interpretability, we assessed the pathways with *p*-values only lower than 5×10^−2^. From the 208 pathways, 58 (28%) had a *p*-value <5×10^−2^ (see Table 3). From these pathways, 44 (76%) can be associated with Marfan disease. From the 58 pathways there are 14 (24%) not known to be associated with Marfan disease, and from these 14 there are 12 pathways that can be associated with the Influenza Virus. This might suggest that Influenza as co-morbidity was present in the patients during data gathering.

**Table 3:** Over representation analysis results of the msPLS analysis on Marfan data

Pathway name	*p*-value	Associated with Marfan disease through pathway
Influenza Virus Induced Apoptosis	3.41 ×10^−5^	Not known*
Non-integrin membrane-ECM interactions	2.92 ×10^−4^	Collagene formation [31]
Anchoring fibril formation	4.73 ×10^−4^	Collagene formation [31]
ECM proteoglycans	6.19 ×10^−4^	Extracellular matrix organization [31]
Integrin cell surface interactions	7.90 ×10^−4^	Extracellular matrix organization [31]
Transcriptional activation of mitochondrial biogenesis	8.17 ×10^−4^	Possibly through reduced mitochondrial respiration [32]
Crosslinking of collagen fibrils	1.20 ×10^−3^	Collagene formation [31]
Laminin interactions	1.98 ×10^−3^	Extracellular matrix organization [31]
Mitochondrial biogenesis	2.40 ×10^−3^	Possibly through reduced mitochondrial respiration [32]
NCAM1 interactions	3.92 ×10^−3^	NCAM signaling for neurite out-growth [33]
Collagen chain trimerization	3.92 ×10^−3^	Collagene biosynthesis and modifying enzymes [31]
TGFBR2 MSI Frameshift Mutants in Cancer	4.20 ×10^−3^	Signaling by TGF-beta receptor complex [31]
Extracellular matrix organization	4.82 ×10^−3^	Extracellular matrix organization [31]
Host Interactions with Influenza Factors	5.02 ×10^−3^	Not known*
Organelle biogenesis and maintenance	5.14 ×10^−3^	Possibly through reduced mitochondrial respiration [32]
Transfer of LPS from LBP carrier to CD14	6.30 ×10^−3^	Possibly through toll-like receptor-4 signaling [34]
Transport of HA trimer, NA tetramer and M2 tetramer from the endoplasmic reticulum to the Golgi Apparatus	6.30 ×10^−3^	Not known*
Loss of Function of TGFBR2 in Cancer	8.39 ×10^−3^	Signaling by TGF-beta receptor complex [31]
TGFBR1 LBD Mutants in Cancer	8.39 ×10^−3^	Signaling by TGF-beta receptor complex [31]
TGFBR2 Kinase Domain Mutants in Cancer	8.39 ×10^−3^	Signaling by TGF-beta receptor complex [31]
Assembly of collagen fibrils and other multimeric structures	8.81 ×10^−3^	Collagene formation [31]
Collagen degradation	9.32 ×10^−3^	Degradation of the extracellular matrix [31]
NCAM signaling for neurite out-growth	9.58 ×10^−3^	NCAM signaling for neurite out-growth [33]
Interleukin-4 and Interleukin-13 signaling	9.78 ×10^−3^	Vascular inflammation through interleukins [35, 36]
Collagen biosynthesis and modifying enzymes	1.12 ×10^−2^	Collagene formation [31]
TGFBR1 KD Mutants in Cancer	1.26 ×10^−2^	Signaling by TGF-beta receptor complex [31]
Loss of Function of TGFBR1 in Cancer	1.46 ×10^−2^	Signaling by TGF-beta receptor complex [31]
SMAD2/3 Phosphorylation Motif Mutants in Cancer	1.46 ×10^−2^	Signaling by TGF-beta receptor complex [31]
Assembly of Viral Components at the Budding Site	1.46 ×10^−2^	Not known*
Loss of Function of SMAD2/3 in Cancer	1.67 ×10^−2^	Signaling by TGF-beta receptor complex [31]
RUNX3 regulates CDKN1A transcription	1.67 ×10^−2^	Signaling by TGF-beta receptor complex [37]
Signaling by TGF-beta Receptor Complex in Cancer	1.88 ×10^−2^	Signaling by TGF-beta receptor complex [31]
Collagen formation	2.02 ×10^−2^	Extracellular matrix organization [31]
Transcriptional regulation of white adipocyte differentiation	2.17 ×10^−2^	Possibly by depleted or abnormal adipose tissue [38]
Aromatic amines can be N-hydroxylated		
or N-dealkylated by CYP1A2	2.29 ×10^−2^	Not known
Formation of annular gap junctions	2.29 ×10^−2^	Endothelial dysfunction [39]
Gap junction degradation	2.50 ×10^−2^	Endothelial dysfunction [39]
Proton-coupled monocarboxylate transport	2.50 ×10^−2^	Not known
RUNX3 regulates p14-ARF	3.31 ×10^−2^	Signaling by TGF-beta receptor complex [37]
Fusion of the Influenza Virion to the Host Cell Endosome	3.52 ×10^−2^	Not known*
Packaging of Eight RNA Segments	3.52 ×10^−2^	Not known*
Fusion and Uncoating of the Influenza Virion	3.72 ×10^−2^	Not known*
Uncoating of the Influenza Virion	3.72 ×10^−2^	Not known*
Budding	3.72 ×10^−2^	Not known*
Release	3.72 ×10^−2^	Not known*
Biosynthesis of protectins	3.72 ×10^−2^	Possibly by proresolving lipid mediators [40]
Degradation of the extracellular matrix	3.87 ×10^−2^	Extracellular matrix organization [31]
RHO GTPases Activate Formins	3.92 ×10^−2^	Extracellular matrix organization [41]
TGF-beta receptor signaling in EMT (epithelial to mesenchymal transition)	3.92 ×10^−2^	Signaling by TGF-beta receptor complex [31]
Cell-extracellular matrix interactions	3.92 ×10^−2^	Extracellular matrix organization [31]
Synthesis of (16-20)-hydroxyeicosatetraenoic acids (HETE)	4.13 ×10^−2^	Arachidonic acid metabolism [42]
Entry of Influenza Virion into Host Cell via Endocytosis	4.13 ×10^−2^	Not known*
Virus Assembly and Release	4.13 ×10^−2^	Not known*
Biosynthesis of maresin-like SPMs	4.33 ×10^−2^	Possibly by proresolving lipid mediators [40]
Biosynthesis of specialized proresolving mediators (SPMs)	4.41 ×10^−2^	Possibly by proresolving lipid mediators [40]
Cytokine Signaling in Immune system	4.49 ×10^−2^	Cytokine signaling [31]
Synthesis of epoxy (EET) and dihydroxyeicosatrienoic acids (DHET)	4.73 ×10^−2^	Arachidonic acid metabolism [42]
Arachidonic acid metabolism	4.76 ×10^−2^	Arachidonic acid metabolism [42]

The pathway names and *p*-values are obtained from https://reactome.org. Not known associations marked with asterisk (*) are all biomolecular pathways associated with reactions to Influenza virus

**Fig. 4:**
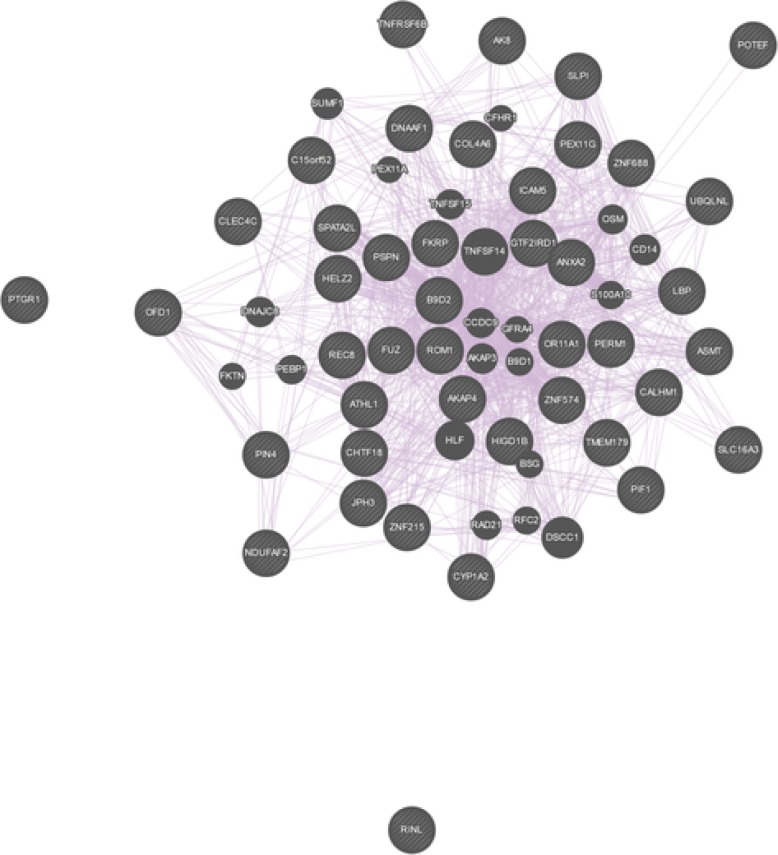
The co-expression pattern of the resulting Marfan genes queried on their biological process based functions. The figure was produced with GeneMania (available at https://genemania.org)

Among the pathways that were identified, already known pathophysiological pathways associated with Marfan disease [31] were found, such as the “*Extracellular matrix organization*” (*p*-value 4.8×10^−3^), the “*Crosslinking of collagen fibrils*” (*p*-value 1.2×10^−3^), the “*TGF-beta receptor signaling in EMT (epithelial to mesenchymal transition)*” (*p*-value 3.92×10^−2^), and the “*Loss of Function of TGFBR2*” (*p*-value 8.39×10^−3^) pathway. The identified pathways can be further appraised in the context of known interactions of genes and genetic phenotypes. We queried the curated database of Online Mendelian Inheritance in Man (OMIM, available at https://www.omim.org). The OMIM query yielded 372 results (the full list can be found in Additional file 3). Among others, OMIM identified the TGF-beta, Collagen IV, Interleukin-6 loci. The identified pathways from these analysis suggest that some patients suffered from Marfan syndrome type 2, which is based on mutations in the TGFBR2 gene (associated pathway “*Loss of Function of TGFBR2*”). The mutation in the FBN1 associated with the classic type of Marfan syndrome. Although MFS2 is phenotypically not separable from classic Marfan syndrome, both disease types include thoracic aortic aneurysm, and more generally aortic risk as the main common feature of the disease [31, 43]. This aortic risk is reportedly caused by the loss of function of extracellular matrix proteins (associated pathway “*Extracellular matrix organization*”), such as collagens and elastin of the vascular wall (associated pathway “*Crosslinking of collagen fibrils*”), that leads to the loss of solidity and elasticity of the blood vessels, including the aorta, ultimately causing thoracic aortic aneurysm. In addition, it has been reported that the activity of transforming growth factor beta (TGF-beta, associated pathway “*TGF-beta receptor signaling in EMT*”), is increased in aneurysmal vascular walls [31, 44–46]. Finally, we examined the physical interaction and co-expression patterns of the list of all genes identified by the first set of LVs (see Table 1) with the online tool GeneMania (available at https://genemania.org). We queried the list of genes based on their biological functions. The analysis resulted in a rich interaction and co-expression pattern (see Fig. 4) with 403 reference studies describing these relationships. The full results of the GeneMania query is available in Additional file 4.

### Chronic lymphocytic leukaemia data

We used msPLS for the simultaneous analysis of 69 genomic, 4248 epigenomic, 5000 transcriptomic and 310 drug response variables measured in 200 chronic lymphocytic leukaemia (CLL) patients. This data is publicly available through the Multi-Omics Factor Analysis (MOFA) R package [11]. We used MOFA to impute the missing variables as described in [11]. A detailed description of this dataset can be found in [29]. The goal of this analysis was to compare msPLS performance in terms of explained variance to the performance of MOFA, a state-of-art unsupervised statistical method for the integrative analysis of multiple omics data sources.

To construct the hierarchical structure between the data sources for the msPLS analysis, we hypothesised the following relationship structure between the data source pairs. We assumed symmetric relationships between the genomic, epigenomic and transcriptomic MVs, and the drug response variables were set as response to both the epigenomic and transcriptomic MVs. We used UST penalisation and we compared our results to the results of MOFA. MOFA’s model selected 5 non-zero biomolecular variables in each LVs. To compare the results to the msPLS, we enforced the model to extract 5 genomic, 30 epigenomic and 30 transcriptomic MVs from the omics sources. Also, we extracted multiple set of LVs per data source, and compared the total captured variation of msPLS’s LVs to MOFA’s LVs. The final model of msPLS resulted in 3 set of LVs that together explained 92% of variance in the genomic variables, 97% of variance in the epigenomic variables, 98% of variance in the transcriptomic variables and 85% of variance in the drug response variables. In comparison, MOFA’s first 10 LVs (i.e. referred to as factors in the MOFA model) together explained 23% of variance in the genomic variables, 24% of variance in the epigenomic variables, 38% of variance in the transcriptomic variables and 40% of variance in the drug response variables (Table 4). We compared the correlations of Table 5 the selected MVs with their corresponding LVs (i.e. these correlations are referred to as loadings in the MOFA model) from msPLS’s and MOFA’s models. The biomarkers extracted with msPLS are listed with their corresponding loadings in Table 6.

**Table 4:** The percentage variation in the chronic lymphocytic leukemia (CLL) data sources explained by the subsequent LVs of msPLS and MOFA

	Genomic variables		Epigenomic variables		Transcriptomic variables		Drug response variables	
	msPLS	MOFA	msPLS	MOFA	msPLS	MOFA	msPLS	MOFA
LV 1	72%	15%	92%	17%	92%	7.5%	57%	15%
LV 2	18%	8.2%	4%	0.5%	5%	4.7%	21%	3.5%
LV 3	2%	<0.1%	1%	<0.1%	1%	1.4%	7%	11.2%
LV 4		<0.1%		<0.1%		9%		<0.1%
LV 5		<0.1%		<0.1%		2.8%		6.1%
LV 6		<0.1%		<0.1%		4.8%		3.4%
LV 7		0.9%		2.4%		1.9%		1%
LV 8		<0.1%		0.5%		3.8%		0.5%
LV 9		<0.1%		2.6%		0.9%		0.4%
LV 10		<0.1%		<0.1%		2.2%		<0.1%
Total	92%	24%	97%	24%	98%	38%	85%	41%

**Table 5:** The weights of the genomic, epigenomic, and transcriptomic variables extracted by msPLS from CLL data sources

Genomic variables		Epigenomic variables		Transcriptomic variables	
Name	**w**	Site	**w**	Gene code	**w**
del11q22.3	0.31	cg06369076	0.036	ADAM29	0.046
del17p13	0.16	cg22449085	0.036	AGPAT4	0.043
BRAF	0.17	cg12208353	0.036	ANK2	0.047
TP53	0.21	cg04694619	0.037	CRY1	0.049
IGHV	-0.66	cg20782816	0.038	DNAH3	0.046
		cg00832703	0.037	ENO4	-0.041
		cg01399475	-0.036	ESPNL	0.043
		cg21398469	0.037	GFI1	0.045
		cg11181763	0.036	GLDN	0.044
		cg01360627	0.036	ITPRIPL2	0.040
		cg09087901	0.036	KANK2	0.047
		cg04848693	0.037	L3MBTL4	0.049
		cg12522599	0.038	LDOC1	0.041
		cg11090458	0.037	LPL	0.041
		cg00148025	0.038	MAPK4	-0.040
		cg12032915	0.036	MRO	0.043
		cg07629149	0.039	MSI2	0.046
		cg23844018	0.037	NDUFA4L2	0.042
		cg05213414	0.037	NUGGC	0.041
		cg01928411	0.037	PLD1	0.043
		cg07699978	0.036	PON1	0.042
		cg03035162	0.036	PRR18	-0.044
		cg03462096	0.039	SEPT10	-0.040
		cg08171667	0.036	SOWAHC	0.041
		cg26441291	0.038	TP63	0.043
		cg21400896	0.037	USP6NL	-0.040
		cg15236196	0.036	VSIG10	0.042
		cg21394039	0.038	ZNF135	-0.040
		cg04613057	0.036	ZNF471	-0.042
		cg08496123	0.036	ZNF667	0.041

**Table 6:** The loadings of the three subsequent LVs extracted by msPLS from the genomic variables of the CLL data set

1st set of LVs		2nd set of LVs		3rd set of LVs	
Name	loading	Name	loading	Name	loading
del11q22.3	0.31	del11q22.3	-0.27	NRAS	0.35
del17p13	0.16	trisomy12	0.65	COL6A5	-0.34
BRAF	0.17	del13q14_any	-0.37	FAM47A	-0.35
TP53	0.21	del14q24.3	0.20	FAT4	-0.39
IGHV	-0.66	CREBBP	0.15	PRPF8	-0.52

**Fig. 5:**
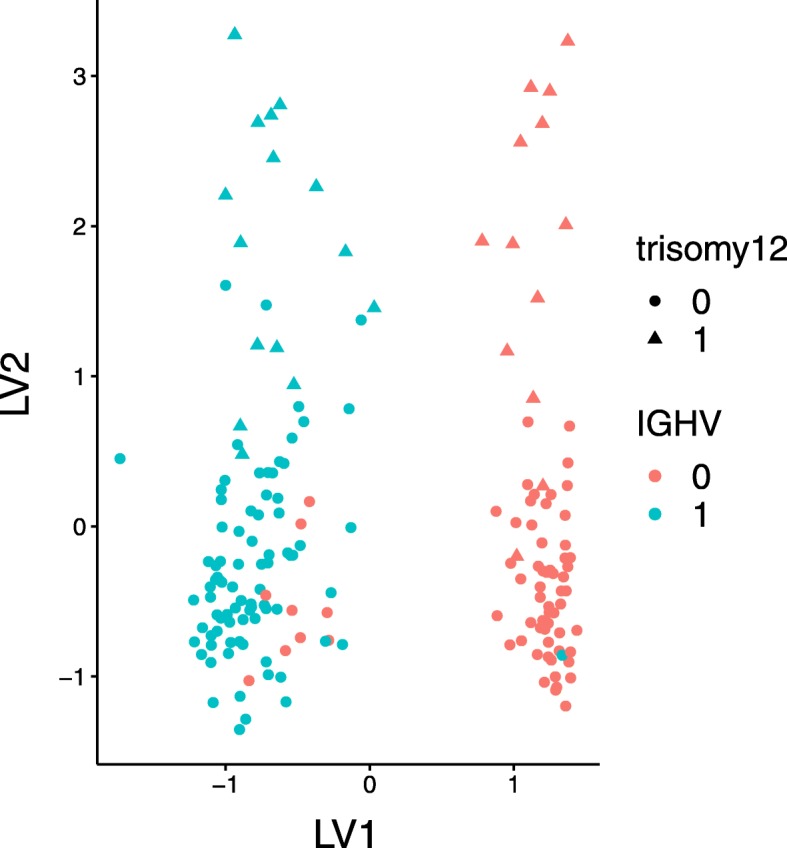
The samples of the CLL data clustered around on their IGHV and trisomy 12 status, extracted by the first and second LV of the msPLS model. The figure was produced by the MOFA R package [11]

We also compared the results of MOFA and msPLS in terms of clinical assessment of the outputs of both models (the full clinical assessment of MOFA’s results can be found in [11]). For this, we used the gene set enrichment analysis in MOFA’s environment. This query resulted in total more than 10,000 pathways, from which 241 pathways with *p*-values <0.05 were identified with the gene sets obtained on the CLL data with MOFA, and 298 pathways with *p*-values <0.05 were identified with the gene sets obtained on the CLL data with msPLS. The first 1000 pathways (ordered by their corresponding *p*-values) for the gene sets from MOFA and msPLS can be found in Additional file 5 and 6. Out of these 1000 pathways, 811 (81%) were identified by both methods, and there are 158 (66% and 53%) overlapping pathways with *p*-values <0.05 (see Additional file 7). Similarly to MOFA, msPLS extracted biomarkers from the genomic variables that can be associated with the pathphysiological pathways of CLL. After querying the gene sets from msPLS, the gene set enrichment analysis identified associations with biological pathways such as the “*Transcriptional regulation of white adipocyte differentiation*” (*p*-value 3.72×10^−4^), the “*Glycerophospholipid biosynthesis*” (*p*-value 5.92×10^−5^), and the “*TP53 Regulates Metabolic Genes*” (*p*-value 4.39×10^−4^) pathway in the first LV. In the second LV, the pathways “*Keratan sulfate/keratin metabolism*” (*p*-value 5.16×10^−5^), “*Post NMDA receptor activation events*” (*p*-value 1.15×10^−4^), and “*Activation of NMDA receptor upon glutamate binding and postsynaptic events*” (*p*-value 2.03×10^−4^) are among the identified ones. Finally, some of the pathways identified in the thirds LV are “*Downstream TCR signaling*” (*p*-value 7.27×10^−71^), “*Translocation of ZAP-70 to Immunological synapse*” (*p*-value 1.52×10^−59^), “*TCR signaling*” (*p*-value 3.14×10^−41^), and “*Immunoregulatory interactions between a Lymphoid and a non-Lymphoid cell*” (*p*-value 8.68×10^−14^). The two most important clinical markers for CLL, namely the immunoglobulin heavy chain gene (IGHV) and the trisomy of chromosome 12 (trisomy12) were extracted as the first and second LV, respectively (Table 6) [11]. Thus similarly to MOFA, the first two set of LVs from msPLS are aligned among IGHV and trisomy12 (the absolute loading of IGHV is 0.66 in the first LV and the absolute loading of trisomy12 is 0.65 in the second LV), and these can be seen as axis of disease heterogeneity. The samples can be clearly clustered based on their IGHV and trisomy 12 status (Fig. 5). Also, there were 140 pathways with *p*-values <0.05 discovered by the gene sets from msPLS that are not overlapping with the pathways discovered by the gene sets from MOFA. Notable pathways that might signal new knowledge discovery are “*Regulation of TP53 Activity through Phosphorylation*” (*p*-value 1.93×10^−4^), “*TP53 Regulates Transcription of Cell Death Genes*” (*p*-value 8.1×10^−4^) [47], “*HDACs deacetylate histones*” (*p*-value 8.73×10^−25^) [48], “*HS-GAG degradation*” (*p*-value 1.22×10^−4^), “*HS-GAG biosynthesis*” (*p*-value 7.6×10^−4^), and “*Heparan sulfate/heparin (HS-GAG) metabolism*” (*p*-value 5.66×10^−3^) [49].

## Discussion

In this paper, we propose a penalised extension of multiset Partial Least Squares path modeling in response to recent reports pointing out the lack of appropriate statistical methods for the simultaneous analysis of multiple high dimensional omics data sources.

msPLS addresses two challenges of integrated high dimensional omics data analysis; namely, it accounts for the relationships between multiple data sources and it provides interpretable results from analyses of hundreds of thousands of biomolecular variables.

Firstly, msPLS accounts for the hierarchical relationship between multiple high dimensional data sources in terms of a explanatory-response dependency structure. It can model dependencies between data sources, such as a hypothesised sequential information transfer in biomolecular domains, through explanatory-response data source pairs. This relationship structure can be easily redefined prior to the analysis, based on most recent biological knowledge. When the relationship is set according to biological knowledge, the biologically relevant biomarkers are identified instead of the variables that explain the most variance in the (combination of) phenotypic variables. Secondly, msPLS provides interpretable results in the form of combinations of biomarkers that have the highest explanatory power for the variance in the phenotypic variables. The biomarkers are extracted along with their weights that indicate their strength of contribution to the overall explained variance. These biomarkers can be further appraised in the context of known biological pathways, for example via gene set enrichment analysis.

Through simulation studies and analyses of omics datasets, we show that msPLS is able to find the combination of biomarkers with the highest explanatory power for the variance in the phenotypic variables, and it can capture a higher proportions of variance in data sources than MOFA, a state-of-art LV based method for multiset omics data analysis. True positive rates of msPLS are reported from the simulation studies (see “Methods” section) to quantify the ability of finding the combination of explanatory variables from the data sources that explain the most variance in response variables. True positive rates range from 0.61 to 0.99, indicating that the precision of finding truly associated variables improves with increasing sample size. Similarly, true negative rates are reported to quantify msPLS’s ability to exclude irrelevant variables from the final model. True negative rates are above 0.99 for each simulation studies, indicating that the final model excludes irrelevant variables with high precision, regardless of sample size.

The analysis of a genomewide omics dataset of 364,134 epigenomic, 18,424 transcriptomic and 47 proteomic variables resulted in biological relevant pathways. msPLS identified a combination of 40 epigenomic biomarkers and 52 transcriptomic biomarkers that has the highest explanatory power for the variance in the phenotypic proteome variables. Despite the low sample size of 37, msPLS identified biomarkers that can be found in known biological pathways associated with the pathophysiology of Marfan disease. Similarly to other LV based multivariate methods, it is possible to extract subsequent LVs with msPLS in a way that they explain a different portion of variance in the data sources. These subsequent LVs are orthogonal to each other, thus the newly obtained biomarkers can be interpreted as biological pathways independent from the ones that were discovered in the previous set of LVs. Comparing the results of msPLS and MOFA on the analyses of the CLL dataset, we found that the three set of LVs from the msPLS model captured 92%, 97%, 98% and 85% of the variation in the genomic, epigenomic, transcriptomic and drug response data sources, respectively, while the first ten LVs of MOFA captured a total of 24%, 24%, 38% and 41% of variation in those same data sources, respectively. msPLS, similarly to MOFA, identified the two most important clinical markers for CLL in its first two LVs, and in the “Results” section we additionally report many highly associated and possible novel pathways found through gene enrichment analysis using the MOFA R package.

Note that the present framework of msPLS assumes linear relationships between data sources and that the omics data is measured on a single homogeneous population. As an interesting future direction to extend msPLS is to incorporate non-linear relations in the model or to extend the model such that it can identify different subgroups in the samples.

## Conclusions

In summary, msPLS is an appropriate multiset multivariate method that can account for the relationships between high dimensional data sources while it provides interpretable results through its sparse solutions. In the “Methods” section we also describe the algorithm for msPLS and we provide an implementation of the algorithm in the open source R software, which is uploaded with the manuscript and available upon request from the authors. We provide open source code that facilitates the use of our msPLS method on new data with the aim to leverage more and more biomolecular data to model and better understand the genetic architectures and biological processes of complex phenotypes, and ultimately to transition the information synthesised from omics data analyses into medical knowledge to improve diagnosis, prognosis and therapy.

## Methods

### Multiset sparse partial least squares path modeling

Multiset sparse Partial Least Squares path modeling (msPLS) is a multivariate approach for modeling the relationship between *Q* related data sources (**X**_1_,...,**X**_*q*_,...,**X**_*Q*_), with the help of latent variables (LVs). Each data source contains *p*_*q*_ number of manifest variables (MVs), measured on the same *n* samples (i.e. $\phantom {\dot {i}\!}\mathbf X_{q} \in \mathbb {R}^{n \times p_{q}}$), each data source is assigned to its corresponding LV (***ζ***_1_,...,***ζ***_*q*_,...,***ζ***_*Q*_). The LVs are linear combinations of their MVs ($\boldsymbol \zeta _{q} = \mathbf X_{q} \mathbf w_{q}\phantom {\dot {i}\!}$, where $\boldsymbol \zeta _{q} \in \mathbb {R}^{n \times 1}$ and $\phantom {\dot {i}\!}\mathbf w_{q} \in \mathbb {R}^{p_{q} \times 1}$). The relationship between the data sources is encoded in a connectivity matrix, like in Partial Least Squares path modeling (PLS-PM), and modelled through a multiple regression model between the LVs;

1 $$ \boldsymbol \zeta_{q} = \sum_{m=1}^{M_{q}} \theta_{qm} \boldsymbol \zeta_{m \rightarrow q} + \mathbf v_{q},  $$

where $\sum _{m=1}^{M_{q}} \boldsymbol \zeta _{m \rightarrow q}$ denotes the sum of *M*_*q*_ LVs that are explanatory for ***ζ***_*q*_, *θ*_*qm*_ is the coefficient capturing the effect of the *m*th ***ζ***_*m*→*q*_ on ***ζ***_*q*_, and **v**_*q*_ is white noise, following the notation of [22, 24] for PLS-PM. A full description for the PLS-PM algorithm can be found in [24] (Algorithm 6). The weight vectors **w**_*q*_ are estimated as

2 $$  \mathbf w_{q} = \left[\mathbf X_{q}^{\prime} \mathbf X_{q}\right]^{-1} \mathbf X_{q}^{\prime} \boldsymbol \zeta_{q},  $$

or as

3 $$ \mathbf w_{q} = (1/n) \mathbf X_{q}^{\prime} \boldsymbol \zeta_{q},  $$

depending on the mode of the regression. PLS-PM denotes Eq. (2) as *Mode A* and Eq. (3) as *Mode B* regression. For msPLS, *Mode A* (i.e. multiple univariate regression) is used for the weight vectors of MVs that do not have any response MVs, and *Mode B* (i.e. multivariate regression) is used for the weight vectors of MVs that do have response MVs. The descriptions of the objective functions of PLS-PM can be found in [22, 24] and the objective function for msPLS is given by Eq. (5) in the “General case” section.

In a high dimensional setting (i.e. *p*_*q*_>>*n*), the covariance matrix of **X**_*q*_ in Eq. (2) is non-invertible. To solve this problem, we propose to replace Eq. (2) with Elastic Net (ENet) penalization. Replacing the ordinary least square estimator in Eq. (2) with ENet penalisation has two advantages; not only we overcome the multicollinearity issue encountered in a high dimensional setting, but ENet also enforces sparse variable selection, which ease the interpretability of the final model. Equation (2) then becomes

4 $$  {}\mathbf w_{q} = arg \underset{\mathbf w_{q} }{\text{min}} \: \mathbf w_{q}^{\prime} \left(\frac{\mathbf X_{q}^{\prime} \mathbf X_{q} + \lambda_{2} \mathbf I}{1 + \lambda_{2}} \right) \mathbf w_{q} - 2 \tilde{\boldsymbol \zeta}_{q}^{\prime} \mathbf X_{q} \mathbf w_{q} + \lambda_{1} \mathbf w_{q},  $$

where *λ*_1_ denotes the LASSO penalty and *λ*_2_ denotes the Ridge penalty parameters [27].

### An example of msPLS with three data sources

**Fig. 6:**
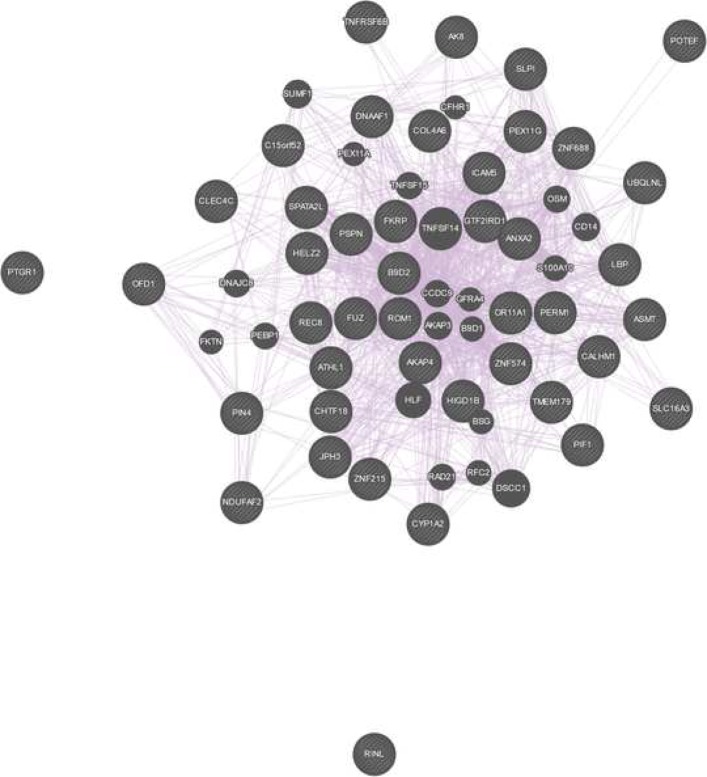
The proposed relationship between three data sources. **X**_1_ and **X**_2_ have a symmetric relation (i.e. they are responses for each other) and **X**_3_ have asymmetric relation with both **X**_1_ and **X**_2_ (i.e. **X**_3_ is response for both **X**_2_ and **X**_1_)

Let us first examine an application of msPLS to three data sources. Given data sources **X**_1_, **X**_2_, and **X**_3_ with *p*_1_, *p*_2_ and *p*_3_ number of variables, measured on *n* samples (i.e. $\phantom {\dot {i}\!}\mathbf X_{1} \in \mathbb {R}^{n \times p_{1}}$, $\phantom {\dot {i}\!}\mathbf X_{2} \in \mathbb {R}^{n \times p_{2}}$ and $\phantom {\dot {i}\!}\mathbf X_{3} \in \mathbb {R}^{n \times p_{3}}$), we consider the following relationships between the data sources: **X**_1_ and **X**_2_ have a symmetric relation (i.e. they are responses for each other). Furthermore, there are asymmetric relations between **X**_1_ and **X**_3_, and between **X**_2_ and **X**_3_, such that **X**_3_ is response for both **X**_2_ and **X**_1_ (Fig. 6). These relationships are encoded in a three dimensional connectivity matrix **C** (i.e. $\phantom {\dot {i}\!}\mathbf {C} \in \{0,1\}^{3 \times 3}$), where the entry $\phantom {\dot {i}\!}c_{{qq}^{\prime }}$ is 1 if data source *q* is response for data source *q*^′^, and 0 otherwise (where *q*≠*q*^′^ and $c_{qq^{\prime }}$ indicates the element from *q*th row and *q*^′^th column of matrix **C**). The objective of the analysis is then to simultaneously extract the MVs from **X**_1_ and **X**_2_ with the highest explanatory power for the variance in MVs of **X**_3_.

**Three data sources msPLS algorithm** Given data sources **X**_1_, **X**_2_, and **X**_3_, and $\boldsymbol \Theta = \mathbf C = \left [\begin {array}{lll} 0 & 1 & 0 \\ 1 & 0 & 0 \\ 1 & 1 & 0 \end {array}\right ]$

1. *Preliminary steps*
  - Center and scale **X**_1_, **X**_2_, and **X**_3_
  - Set $\mathbf w_{1}^{(0)}$, $\mathbf w_{2}^{(0)} $ and $\mathbf w_{3}^{(0)}$ initial weight vectors to arbitrary vectors of [1,1,...,1]^′^ with length *p*_1_, *p*_2_ and *p*_3_, respectively
  - Define convergence criterion *CRT*=1 and a small positive tolerance *γ*=10^−6^
2. *Iterative regression steps*
  While *CRT*≥*γ*;
  a. *Estimate initial LVs*$\boldsymbol \zeta _{1} \propto \mathbf X_{1} \mathbf w_{1}^{(0)} $; where ∝ indicates that ***ζ***_1_ is normalised to unit variance
  $\boldsymbol \zeta _{2} \propto \mathbf X_{2} \mathbf w_{2}^{(0)} $
  $\boldsymbol \zeta _{3} \propto \mathbf X_{3} \mathbf w_{3}^{(0)} $
  b. *Model the relationship between data sources*
  (i) Let vector **c**_*q*_ be the *q*-th row of **C** that indicates the data sources that are explanatory for data source *q*, i.e. **c**_1_=[0,1,0],**c**_2_=[1,0,0],**c**_3_=[1,1,0]; indicating **X**_1_ has one explanatory, **X**_2_ has one explanatory and **X**_3_ has two explanatory data sources
  **If**${\sum ^{3}_{i=1} c_{qi}} { > 0}$, i.e. if data source *q* has any explanatory data sources:
  $\mathbf \Theta _{\mathbf c_{q} q} = \left [\mathbf Z_{\mathbf c_{q}}^{\prime } \mathbf Z_{\mathbf c_{q}}\right ]^{-1} \mathbf Z_{\mathbf c_{q}}^{\prime } \boldsymbol \zeta _{q},$
    where **Z** is the matrix of column bind LVs, i.e. **Z**=[***ζ***_1_,***ζ***_2_,***ζ***_3_], and $\mathbf Z_{\mathbf c_{q}}$ is the matrix of column bind explanatory LVs of data source *q*. Then $\mathbf \theta _{\mathbf c_{q} q}$ is calculated as follows:
    For **c**_1_ we calculate
    $\mathbf \Theta _{\mathbf {c}_{1} 1} = \theta _{2 1} = \left [\boldsymbol \zeta _{2}^{\prime } \boldsymbol \zeta _{2}\right ]^{-1} \boldsymbol \zeta _{2}^{\prime } \boldsymbol \zeta _{1}$ and the value of *θ*_11_ and *θ*_31_ remain 0.
    For **c**_2_ we calculate
    $\mathbf \Theta _{\mathbf c_{2} 2} = \theta _{1 2} = [\boldsymbol \zeta _{1}^{\prime } \boldsymbol \zeta _{1}]^{-1} \boldsymbol \zeta _{1}^{\prime } \boldsymbol \zeta _{2}, $ and the value of *θ*_22_ and *θ*_32_ remain 0,
    and for **c**_3_ we calculate
    $\mathbf \Theta _{\mathbf c_{3} 3} = \begin {array}{l} \theta _{1 3}\\ \theta _{2 3} \end {array}^{\prime } = \big [[\boldsymbol \zeta _{1}, \boldsymbol \zeta _{2}]^{\prime } [\boldsymbol \zeta _{1}, \boldsymbol \zeta _{2}]\big ]^{-1} [\boldsymbol \zeta _{1}, \boldsymbol \zeta _{2}]^{\prime } \boldsymbol \zeta _{3},$
    where the entries *θ*_13_ and *θ*_23_ are obtained from the multiple regression step [[***ζ***_1_,***ζ***_2_]^′^[***ζ***_1_,***ζ***_2_]]^−1^[***ζ***_1_,***ζ***_2_]^′^***ζ***_3_, and [***ζ***_1_,***ζ***_2_] is the matrix obtained by column binding ***ζ***_1_ and ***ζ***_2_. The value of *θ*_33_ remains 0.
  (ii) Let vector $\mathbf c_{q^{\prime }}\phantom {\dot {i}\!}$ be the *q*^′^-th column of **C** that indicates the data sources that are response for data source *q*^′^, i.e. **c**_1_=[0,1,1]^′^,**c**_2_=[1,0,1]^′^,**c**_3_=[0,0,0]^′^; indicating **X**^1^ has two responses, **X**_2_ has two responses and **X**_3_ has no response data sources
  **If**${\sum ^{3}_{i=1} c_{iq^{\prime }}} { > 0}$, i.e. if data source *q*^′^ has any response data sources:
  $\boldsymbol \Theta _{\mathbf c_{q^{\prime }} q^{\prime }} = cor(\boldsymbol \zeta _{q^{\prime }},\boldsymbol \zeta _{\mathbf c_{q^{\prime }}}),$
    i.e., for **c**_1_ we calculate
    $\mathbf {\Theta }_{\mathbf c_1 1} = \begin {array}{l} \theta _{2 1}\\ \theta _{3 1} \end {array} = \begin {array} cor(\boldsymbol \zeta _{1},\boldsymbol \zeta _{2})\\ cor(\boldsymbol \zeta _{1},\boldsymbol \zeta _{3}) \end {array}$
    and for **c**_2_ we calculate
    $\mathbf \Theta _{\mathbf c_2 2} = \begin {array}{l} \theta _{1 2}\\ \theta _{3 2} \end {array} = \begin {array}{l} cor(\boldsymbol \zeta _{2},\boldsymbol \zeta _{1})\\ cor(\boldsymbol \zeta _{2},\boldsymbol \zeta _{3}) \end {array}$
    After Steps (b-i) and (b-ii), the entries of **Θ** are;
    $$\mathbf \Theta =\left[ \begin{array}{ccc} 0 & cor(\boldsymbol{\zeta_{2}},\boldsymbol{\zeta_{1}}) & \theta_{13} \\ cor(\boldsymbol \zeta_{1},\boldsymbol \zeta_{\mathbf 2}) & 0 & \theta_{23} \\ cor(\boldsymbol \zeta_{1},\boldsymbol \zeta_{\mathbf 3}) & cor(\boldsymbol \zeta_{2},\boldsymbol \zeta_{\mathbf 3}) & 0 \end{array}\right],$$
    Notice that *θ*_21_ and *θ*_12_ in Step (b-i) are overwritten in Step (b-ii). This is because ***ζ***_1_ and ***ζ***_2_ are both responses to each other.
  c. *Re-estimate the the latent variables*
  $[\tilde {\boldsymbol \zeta }_{1},\tilde {\boldsymbol \zeta }_{2}, \tilde {\boldsymbol \zeta }_{3}] = [\boldsymbol \zeta _{1},\boldsymbol \zeta _{2}, \boldsymbol \zeta _{3}] \mathbf \Theta $
  d. *Estimate the new ***w**^(1)^ weights
  $ \mathbf w_{1}^{(1)} = arg \underset {\mathbf w_{1}^{(0)}}{\text {min}} \: \mathbf w_{1}^{'(0)} \bigg (\frac {\mathbf X_{1}^{\prime } \mathbf X_{1} + \lambda _{2} \mathbf I}{1 + \lambda _{2}}\bigg) \mathbf w_{1}^{(0)} - 2 \tilde {\boldsymbol \zeta }_{1}^{\prime } \mathbf X_{1} \mathbf w_{1}^{(0)} + \lambda _{1} \mathbf w_{1}^{(0)}$
  $ \mathbf w_{2}^{(1)} = arg \underset {\mathbf w_{2}^{(0)}}{\text {min}} \: \mathbf w_{2}^{'(0)} \bigg (\frac {\mathbf X_{2}^{\prime } \mathbf X_{2} + \lambda _{2} \mathbf I}{1 + \lambda _{2}}\bigg) \mathbf w_{2}^{(0)} - 2 \tilde {\boldsymbol \zeta }_{2}^{\prime } \mathbf X_{2} \mathbf w_{2}^{(0)} + \lambda _{1} \mathbf w_{2}^{(0)}$
  $ \mathbf w_{3}^{(1)} = \big [ [\tilde {\boldsymbol \zeta }_{3}^{\prime } \tilde {\boldsymbol \zeta }_{3}]^{-1} \tilde {\boldsymbol \zeta }_{3}^{\prime } \mathbf X_{3} \big ]^{\prime }$
  e. *Evaluate the convergence criteria and discard the old* **w**^(0)^ weights
  $CRT = \sum ^{3}_{q=1} (\mathbf w_{q}^{(1)}-\mathbf w_{q}^{(0)})^{2}$
  $\mathbf w_{1}^{(0)} = \mathbf w_{1}^{(1)}$, $\mathbf w_{2}^{(0)} = \mathbf w_{2}^{(1)}$ and $\mathbf w_{3}^{(0)} = \mathbf w_{3}^{(1)}$
3. *Upon convergence, return*$\mathbf w_{1}^{(0)},\mathbf w_{2}^{(0)}$, *and*$\mathbf w_{3}^{(0)}$

### General case

The general case for msPLS can be described as follows. Given *Q* related data sources **X**_1_,...,**X**_*q*_,...,**X**_*Q*_ with *p*_1_,...,*p*_*q*_,...*p*_*Q*_ corresponding MVs, measured on *n* samples (i.e. $\phantom {\dot {i}\!}\mathbf {X}_1 \in \mathbb {R}^{n \times p_1}$,..., $\phantom {\dot {i}\!}\mathbf {X}_q \in \mathbb {R}^{n \times p_q},...,\mathbf {X}_Q \in \mathbb {R}^{n \times p_Q}$), and a *Q* dimensional connectivity matrix **C** (i.e. **C**∈{0,1}^*Q*×*Q*^), where the entry $\phantom {\dot {i}\!}c_{{qq}^{\prime }}$ is 1 if data source *q* is a response data source for data source *q*^′^ and 0 otherwise. The goal of the analysis then is to optimise the following objective function () in respect to data source *q*^′^;

5 $$  OF \,=\, arg\text{max}\! \left\{\begin{array}{ll} \sum_{r=1}^{R_{q^{\prime}}} Cor (\boldsymbol \zeta_{q^{\prime} \rightarrow r},\boldsymbol \zeta_{q^{\prime}})^{2} & \text{if}\ {\sum^{Q}_{i=1} c_{iq^{\prime}}} { > 0} \\ \sum_{i=1}^{p_{q^{\prime}}} \sum_{m=1}^{M_{q^{\prime}}} Cor (\boldsymbol \zeta_{m \rightarrow q^{\prime}},\mathbf x_{q^{\prime}(i)})^{2} & \text{{otherwise}} \\ \end{array}\right.  $$

where ***ζ***_*q*_ is the LV of $\phantom {\dot {i}\!}\mathbf X_{q'}$, $\phantom {\dot {i}\!}\mathbf c_{q'}$ indicates the *q*^′^th column of matrix **C** (i.e. $\phantom {\dot {i}\!}|| \mathbf c_{q'}|| > 0$ indicates that data source *q*^′^ have at least one response data source), $\mathbf x_{q'(i)}\phantom {\dot {i}\!}$ denotes the *i*th column of data source $\phantom {\dot {i}\!}\mathbf X_{q'}$ (i.e. the *i*th MV of $\phantom {\dot {i}\!}\mathbf X_{q'}$), $\phantom {\dot {i}\!}\sum _{r=1}^{R_{q'}} \boldsymbol \zeta _{q' \rightarrow r}$ denotes the sum of $\phantom {\dot {i}\!}R_{q'}$ LVs that are response for $\phantom {\dot {i}\!}\boldsymbol \zeta _{q'}$, and $\sum _{m=1}^{M_{q'}} \boldsymbol \zeta _{m \rightarrow q'}\phantom {\dot {i}\!}$ denotes the sum of $\phantom {\dot {i}\!}M_{q'}$ LVs that are explanatory for $\phantom {\dot {i}\!}\boldsymbol \zeta _{q'}$. In other words, if data source *q*^′^ have at least one response data source, then the squared correlation between $\phantom {\dot {i}\!}\boldsymbol \zeta _{q'}$ and the combination of its response LVs is maximised, and if data source *q*^′^ does not have any response data sources, the correlation between the MVs of $\mathbf {X}_{q^{\prime }}\phantom {\dot {i}\!}$ and the combination of the explanatory LVs for $\phantom {\dot {i}\!}\mathbf {X}_{q^{\prime }}$ is maximised. The symmetric relationship between **X**_*q*_ and $\mathbf {X}_{q^{\prime }}\phantom {\dot {i}\!}$ is indicated as $\phantom {\dot {i}\!}c_{{qq}^{\prime }} = c_{q^{\prime } q} = 1$, in which case the OF of their pairwise analysis is to maximise the correlation between their LVs ***ζ***_*q*_ and $\boldsymbol \zeta _{q^{\prime }}\phantom {\dot {i}\!}$, corresponding to the characteristic objective function of Canonical Correlation Analysis (CCA) [8, 22, 50]. In an asymmetric relationship, the OF of a pairwise analysis is to maximise the sum of squared correlation between the explanatory LV ***ζ***_*q*_ and the response MVs in $\mathbf {X}_{q^{\prime }}\phantom {\dot {i}\!}$, corresponding to the characteristic objective function of Redundancy Analysis (RDA) [10, 22, 51]. This direct correspondence with CCA and RDA is described in Additional file 1 under the Modes of relationships between data sources section.

Next we describe the general algorithm for *Q* data sources.

**General msPLS algorithm**

- Given *Q* data sources **X**_1_,..,**X**_*q*_,...,**X**_*Q*_,
- and **Θ**=**C**∈{0,1}^*Q*×*Q*^, where $c_{q,q'} = \left \{\begin {array}{ll} 1 & \text { if } \mathbf X_{q} \text { response for } \mathbf X_{q^{\prime }}\\ 0 & {otherwise} \end {array}\right.$

1. *Preliminary steps*
  - Center and scale **X**_1_,..,**X**_*q*_,...,**X**_*Q*_
  - Set $\mathbf w_{q}^{(0)}$ to arbitrary weight vectors [1,1,...,1]^′^ with length *p*_*q*_
  - Define convergence criterion *CRT*=1 and a small positive tolerance *γ*=10^−6^
2. *Iterative regression steps*
  While *CRT*≥*γ*;
  a. *Estimate initial LVs*
  $\boldsymbol \zeta _{q} \propto \mathbf X_{q} \mathbf w_{q}^{(0)} $; where *q* is the index from 1 to *Q* and ∝ indicates that ***ζ***_*q*_ is normalised to unit variance
  b. *Model the relationship between data sources*
  (i) Let vector **c**_*q*_ be the *q*-th row of **C** that indicates the data sources that are exploratory for data source *q*
  **If**${\sum ^{Q}_{i=1} c_{qi}} { > 0}$, i.e. if data source *q* has any explanatory data sources:
  $ \mathbf \Theta _{\mathbf c_{q} q} = [\mathbf Z_{\mathbf c_{q}}^{\prime } \mathbf Z_{\mathbf c_{q}}]^{-1} \mathbf Z_{\mathbf c_{q}}^{\prime } \boldsymbol \zeta _{q},$
    where **Z** is the matrix of column bind LVs, i.e. **Z**=[***ζ***_1_,***ζ***_2_,***ζ***_3_], and $\mathbf Z_{\mathbf c_{q}}$ is the matrix of the column-bind explanatory LVs of data source *q*.
  (ii) Let vector $\mathbf c_{q'}\phantom {\dot {i}\!}$ be the *q*^′^-th column of **C** that indicates the data sources that are response for data source *q*^′^
  **If**$\phantom {\dot {i}\!}{\sum ^{Q}_{i=1} c_{iq^{\prime }}} { > 0}$, i.e. if data source *q*^′^ has any responses:
  $\phantom {\dot {i}\!}\boldsymbol \Theta _{\mathbf c_{q^{\prime }}q^{\prime }} = cor(\boldsymbol \zeta _{q^{\prime }},\boldsymbol \zeta _{\mathbf c_{q^{\prime }}})$
  c. *Re-estimate the LVs*
  $\tilde {\mathbf Z} = \mathbf Z \mathbf \Theta $
  d. *Estimate the new *$\mathbf w_{q}^{(1)}$ weights
  **If** **X**_*q*_ doesn’t have any response data sources:
  $ \mathbf w_{q}^{(1)} = \big [ [\tilde {\boldsymbol \zeta }_{q}^{\prime } \tilde {\boldsymbol \zeta }_{q}]^{-1} \tilde {\boldsymbol \zeta }_{q}^{\prime } \mathbf X_{q} \big ]^{\prime } $
  **otherwise**:
  $\mathbf w_{q}^{(1)} = arg \underset {\mathbf w_{q}^{(0)} }{\text {min}} \: \mathbf w_{q}^{'(0)} \bigg (\frac {\mathbf X_{q}^{\prime } \mathbf X_{q} + \lambda _{2} \mathbf I}{1 + \lambda _{2}}\bigg)\mathbf w_{q}^{(0)} - 2 \tilde {\boldsymbol \zeta }_{q}^{\prime } \mathbf X_{q} \mathbf w_{q}^{(0)} + \lambda _{1} \mathbf w_{q}^{(0)}$
  e. *Evaluate the convergence criteria and discard the old*$\mathbf w_{q}^{(0)}$*weights and calculate OF from Eq.* (5) *with respect to each data sources*
  $CRT = \sum (\mathbf w_{q}^{(1)}- \mathbf w_{q}^{(0)})^{2}$
  $\mathbf w_{q}^{(0)} = \mathbf w_{q}^{(1)}$
3. Upon convergence, return $\mathbf w_{q}^{(0)}$

After the algorithm converges, the *w*_*q*_ weights indicate the contribution of explanatory MVs from the *q*th data source towards the overall explained variance in the response MVs or LVs (see Additional file 1 under Modes of relationships between data sources section). Through the penalisation of the multivariate regression in Step (2-d), a small subset of explanatory MVs are extracted, namely those with the highest explanatory power for the variance in their response MVs or LVs. The extracted set of MVs can be further explored in terms of known biological pathways, for example through gene enrichment analysis.

### Multiple latent variables per dataset

It is possible to extract multiple LVs per data source in a way that they explain a different portion of variance in the MVs. The explained variance is based on the *R*^2^ statistic obtained from the regression model from Step (2-d) in the general msPLS algorithm. The subsequent latent variables can be obtained by applying msPLS to the residual data sources, where the residuals data sources are calculated as

$$\begin{aligned} \mathbf{X}_{q(\alpha)}^{res} &= \mathbf{X}_{q(\alpha)} - \hat{\mathbf{X}}_{q(\alpha)}\\ &= \mathbf{X}_{q(\alpha)} - \boldsymbol \zeta_{q(\alpha)} [\boldsymbol \zeta_{q(\alpha)}^{\prime} \boldsymbol \zeta_{q(\alpha)}]^{-1} \boldsymbol \zeta_{q(\alpha)}^{\prime} \mathbf{X}_{q(\alpha)}. \end{aligned} $$

### Selecting the optimal penalisation parameters and assessing the statistical significance of the resulting model

In order to obtain the **w**_*q*_ weights that optimise OF in Eq. (5), the optimal LASSO and Ridge penalisation parameters can be selected through *k*-fold cross validation. Given the usual size of omics data and the multiset approach of the analysis, searching for the optimal penalisation parameters is often too computationally expensive. As a solution, we propose to use Univariate Soft Thresholding (UST), by setting *λ*_2_→*∞* in Eq. (4) [27].

To assess the statistical significance of a resulting model in respect to the OF in Eq. (5), we use a standard permutation approach. The null distribution of the optimisation criterion is estimated by applying msPLS to permuted dataset, where we permute the rows of each dataset. Permuting the samples removes the correlation between data sources while the internal correlation structure of each data source is preserved. The weights obtained from the permutation are used to calculate OF, and the null distribution of the optimisation criterion can be approximated by repeating the permutation a large number of times. In addition, we use bootstrapping to approximate the confidence intervals for the optimised OF. During bootstrapping, the observations are sampled with replacement and the penalisation parameters from the original model are used for the bootstrap samples. In contrast to permutation, with bootstrapping the correlation between data sources is also preserved. After repeating the bootstrapping many times, the selected quantiles of the resulting distribution are reported.

### Assessing msPLS’s ability to identify associated variables among multiple high dimensional data sources

Before we applied msPLS to omics data sources, we analysed simulated data to assess msPLS’s ability to extract the associated MVs from multiple high dimensional data sources that optimise the OF in Eq. (5). Then we applied msPLS to omics data sources to see whether the resulting model can be interpreted in terms of known biological pathways. Below, we describe the simulation studies, and the real data analysis can be found in the “Results” section.

#### Simulation studies

We conducted simulation studies to assess msPLS’s ability to identify associated MVs (i.e. explanatory MVs that are highly correlated with their response MVs and thus have the highest explanatory power for the variance in the response MVs) when those MVs are spread over multiple data sources. We repeated the simulations 1000 times and used UST penalisation for which the optimal penalty parameter (*λ*_1_) was selected through 10-fold cross validation. Additionally, we assessed the statistical significance of the resulting models through permutations and the confidence interval of the optimisation criterion was approximated through bootstrapping.

#### Data generation for simulation studies

For all simulation studies, we generated three data sources, **X**_1_, **X**_2_ and **X**_3_, in such way that the relationship between data sources resembles the one we describe in “Chronic lymphocytic leukaemia data” section (Fig. 4).

All **X**_*q*_s were assigned to *p*_*q*_ number of MVs (i.e. *p*_1_ = *p*_2_ = 1000, *p*_3_ = 100) from which *k*_*q*_ variables were associated with their LVs and response MVs (i.e. *k*_1_ = *k*_2_ = *k*_3_ = 10), and there were *j*_*q*_ number of not associated MVs (i.e. *j*_1_ = 990, *j*_2_ = 990, *j*_3_ = 90). The number of samples are denoted by *n* samples (i.e. $\mathbf X_q \in \mathbb {R}^{n \times p_{q}}$ with *k*_*q*_ associated MVs and *j*_*q*_ not associated MVs, *p*_*q*_ = *k*_*q*_ + *j*_*q*_), and in the first three simulation studies *n* varied from 1, 100, and 250.

**X**_1_ and **X**_2_ were generated from a multivariate normal distribution with mean 0 and covariance matrix **Σ**, and their response MVs in **X**_3_ was generated from LVs ***ζ***_1_ and ***ζ***_2_, as follows;

1. **Σ**=**I**_2000_
2. Replace $\mathbf \Sigma _{1001 : 1010, 1 : 10} = \mathbf \Sigma _{1:10, 1001:1010}^{'} = \mathbf H$,
  where $\mathbf H \in \mathbb {R}^{10 \times 10}$ distributed over $\mathcal {N}(0.3,0.05)$
3. $\mathbf D \sim \mathcal {N}(0,\mathbf \Sigma)$ where $\mathbf D \in \mathbb {R}^{n \times 2000}$
4. **X**_1_=**D**_1:*n*,1:1000_ and **X**_2_=**D**_1:*n*,1001:2000_

**Σ** is a *p*_1_ + *p*_2_ dimensional identity matrix where elements $\mathbf \Sigma _{1001 : 1010,1 : 10} = \mathbf \Sigma _{1:10, 1001:1010}^{'}$ were replaced with **H**, where $\mathbf H \in \mathbb {R}^{10 \times 10}$ was distributed over $\mathcal {N}(0.3,0.05)$. **D** was sampled from the multivariate normal distribution with mean 0 and covariance matrix **Σ**, and **D** was used to generate **X**_1_ and **X**_2_. Next, the weight vectors were generated;

(5) $\mathbf w_q = (w_{q(1)}, w_{q(2)},..., w_{q (k_1)}, w_{q (k_{1}+1)},... w_{q (p_1)}\phantom {\dot {i}\!}$), $\mathbf w_{q(1:k_1)} = w_{q}^{associated}\phantom {\dot {i}\!}$, $\mathbf w_{q (k_{q}+1:p_q)} = 0\phantom {\dot {i}\!}$

The associated *k* MVs had higher regression weights with their LVs (with weights $\phantom {\dot {i}\!}w_{1}^{associated}$ = 0.7, $\phantom {\dot {i}\!}w_{2}^{associated}$ = 0.6, $w_{3}^{associated}$ = 0.3) than the not associated *j*_*q*_ MVs (i.e. $\mathbf w_q = (w_{q1}, w_{q2},..., w_{q k_q}, w_{q k_{q}+1},... w_{q p_q}\phantom {\dot {i}\!}$), $\phantom {\dot {i}\!}\mathbf w_{q (1:k_q)} = w_{q}^{associated}$, $\mathbf w_{q (k_{q}+1:p_q)} = 0\phantom {\dot {i}\!}$). The LVs were generated as a linear combination of the MVs and weights,

(6) ***ζ***_***1***_=**X**_1_**w**_1_ and ***ζ***_2_=**X**_2_**w**_2_

**X**_3_ was generated with from ***ζ***_1_ and ***ζ***_2_. The *k*_3_ associated LVs were sampled from the normal distribution with mean *θ*_1_*ζ*_1_ + *θ*_2_*ζ*_2_ (where *θ*_*q*_ is the regression coefficient from Eq. (1), with *θ*_1_ = 0.8 and *θ*_2_ = 0.7) and standard deviation $\sqrt []{1- (w_3)^{2}}$. The *j*_3_ not associated variables were sampled from the standard normal distribution;

(7) $\mathbf X_3 \in \mathbb {R}^{n \times 100}$
(8) For *i*=1,...,*k*_3_:
  **X**_3(*i*)_ distributed $\mathcal {N}(\theta _1 \zeta _1 + \theta _2 \zeta _2, \sqrt []{1- (w_3)^{2}})$
(9) For *i*=*k*_3_+1,...,*p*_3_:
  **X**_3(*i*)_ distributed $\mathcal {N}(0, 1)$

In addition, we designed a fourth simulation study, where the size of the data resambled the size of the omics data sources, described in “Results” section (i.e. *p*_1_ = 360000, *p*_2_ = 18000, *p*_3_ = 47, *k*_1_ = *k*_2_ = 40, *k*_3_ = 10, and *n* = 37).

#### Simulation study results

We generated data as described in above with three different sample sizes, i.e. *n*=50, *n*=100, *n*=250. To assess msPLS’s ability to identify the *k*_*q*_ associated MVs from explanatory data sources **X**_1_ and **X**_2_, we used the true-positive rate (TPR) and true-negative rate (TNR) measures over 1000 simulations.

TPR measures the proportion of associated MVs included in the final model (i.e. those that are assigned to non-zero **w** weights) to either the number of associated MVs that were generated, or to the total number of non-zero **w** weights, whichever is smaller (i.e. $TPR_q = \sum _{i=1}^{k_q}I(w_{q(i)} \neq 0) / {\text {min}}(k_q, \sum _{i=1}^{p_q}I(w_{q(i)} \neq 0))$). TPR ranges from 0.61 to 0.99 and increases with increasing sample size when the variable size held constant (Table 7).

**Table 7:** True-positive rate (TPR) and true-negative rate (TNR) results of the simulation study

	n = 50	n = 100	n = 250	n = 37
$TPR_{X_1}$	0.67	0.93	0.99	0.61
$TPR_{X_2}$	0.66	0.94	0.99	0.72
$TNR_{X_1}$	0.99	0.99	0.99	0.99
$TNR_{X_2}$	0.99	0.99	0.99	0.99

TNR measures the proportion of not associated MVs excluded from the model to the number of not associated MVs that were generated (i.e. $TNR_q = \sum _{i=k_{q}+1}^{p_q}I(w_{q(i)} = 0) / j_{q}$). TNR rates resulted in 0.99 and were not affected by the sample size (Table 7).

**Fig. 7:**
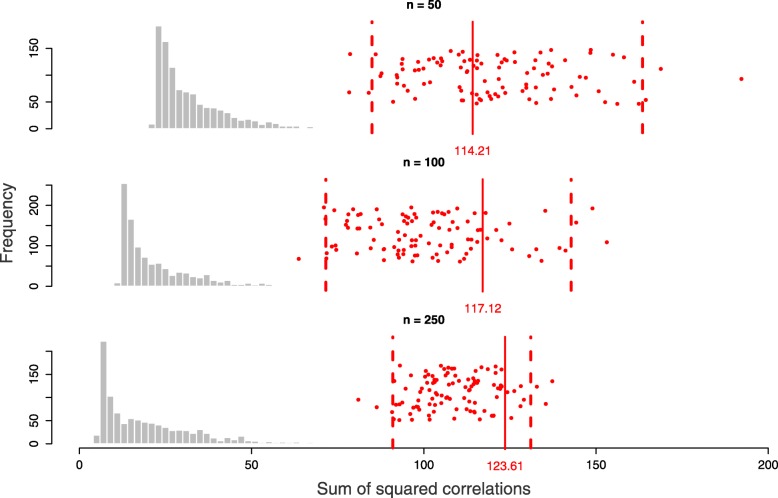
The null distributions of the optimisation criteria (with respect to **X**_3_) for the simulated data with different sample sizes (n = 50, 100, 250), obtained after 1000 permutations. The red bars indicate the optimisation criteria obtained applying msPLS to the original data with the optimal *λ*_1_ parameters for UST. The red dots are the bootstrapped values, and the dashed red bars are the 95% confidence intervals

We assessed the statistical significance of the resulting models in respect to the optimised OFs through permutation, and the confidence intervals of the optimised OFs were constructed through bootstrapping (see the “Selecting the optimal penalisation parameters and assessing the statistical significance of the resulting model” section). All the three models obtained on the three different sample sizes with constant variable size were statistically significant, and the confidence interval of the optimised OFs shrank with increased sample size (Fig. 7). For *n*=50, the optimised OF with respect to **X**_3_ resulted in 114.21 (95% CI [85.02, 163.43], *p*-value <0.001), for *n*=100 the optimised OF resulted in 117.12 (95% CI [71.58, 142.69], *p*-value <0.001), and for *n*=250 the optimised OF resulted in 123.61 (95% CI [91.04, 130.94], *p*-value <0.001).

## Availability and Requirements

Project name: msPLS implementation

Project home page: http://uva.csala.me/mspls and https://github.com/acsala/2018_msPLS

Operating system(s): Platform independent

Programming language: R

Other requirements: additional R packages listed in the source code, freely available from the Comprehensive R Archive Network online

License: MIT

Any restrictions to use by non-academics: MIT license applies

## Supplementary information

**Additional file 1** Appendix: Supplementary materials for the proposed method and results in pdf file format.

**Additional file 2** Supplementary materials for the gene set enrichment analysis results of the Marfan data, obtained from Reactome in coma separated file format.

**Additional file 3** Supplementary OMIM Gene Map Retrieval results of the Marfan data, obtained from OMIM in xls file format.

**Additional file 4** Supplementary co-expression pattern results of the Marfan data, obtained from Gene Mania in txt file format.

**Additional file 5** Supplementary gene set enrichment analysis results of the Chronic lymphocytic leukaemia data analyzed by MOFA. Results are obtained by the MOFA R package and exported as txt file format.

**Additional file 6** Supplementary gene set enrichment analysis results of the Chronic lymphocytic leukaemia data analyzed by msPLS. Results are obtained by the MOFA R package and exported as txt file format.

**Additional file 7** Supplementary material on the overlapping gene set enrichment analysis results between msPLS and MOFA on the Chronic lymphocytic leukaemia data. Results are obtained by the MOFA R package and exported as txt file format.

## Data Availability

An R implementation of msPLS that contains instructions for data simulation and analysis is available at http://uva.csala.me/mspls. The Marfan data analysed during the current study are not publicly available due to privacy reasons, and the Chronic lymphocytic leukaemia data are available in the MOFA R package, https://github.com/bioFAM/MOFA.

## Abbreviations

CCA: Canonical correlation analysis
CCL: Chronic lymphocytic leukaemia
CI: Confidence interval
ENeT: Elastic net
LVs: Latent variables
LASSO: Least absolute shrinkage and selection operator
MVs: Manifest variables
MOFA: Multi-omics factor analysis
msPLS: Multiset sparse partial least squares path modeling
OF: Objective function
OMIM: Online mendelian inheritance in man
PLS-PM: Partial least squares path modeling
RDA: Redundancy analysis
TNR: True-negative rate
TPR: True-positive rate
UST: Univariate soft thresholding
