# Supplementary material for: Multiset sparse partial least squares path modeling for high dimensional omics data analysis
Source: BMC Bioinformatics. 2020 Jan 9;21:9. doi: 10.1186/s12859-019-3286-3 (PMC6953292; doi:10.1186/s12859-019-3286-3)
Supplement: Supplementary file 1 — Additional file 1 Appendix: Supplementary materials for the proposed method and results in pdf file format. [file 12859_2019_3286_MOESM1_ESM.pdf]

Appendix

Modes of relationships between data sources

msPLS is able to account for relationship between multiple data sources. The possible relationship forms between each data source pair A and B is that either they are not connected, or they are connected through a symmetrical or an asymmetrical relationship.

Symmetric and asymmetric relationship between data sources

We consider three cases to model the relationship between data source pairs. In the first case, data source A can be considered as explanatory to data source B (and therefore data source B as response for data source A), so that the MVs in data source A will be regarded as explanatory for MVs in data source B. In the second case, data source B can be considered as explanatory to data source A. These first two cases are called asymmetric relationships between data sources. In the third case, data source A can be considered as response to data source B while data source B is also considered as response for data source A. This third case is called a symmetrical relationship. For msPLS, we will restrict the relationships between two data sources to these three cases. The reason to not include all the relationship models proposed for PLS-PM (see [1] for an overview of all possible relationship models) and restrict msPLS the above three is that they correspond to well known multivarate methods and therefore they have well defined objective functions regarding the optimisation criterion of the analysis. We show in the next sections that in the case when msPLS is applied to two data sources and the data sources have a symmetrical relationship corresponds to Canonical Correlation Analysis (CCA) and that an asymmetric relationship corresponds to Redundancy Analysis (RDA).

Asymmetric relationship of two data sources: RDA

msPLS two data sources example

Given data sources $\mathbf{X}_1$, $\mathbf{X}_2$

(i) *Preliminary steps*

- Center and scale $\mathbf{X}_1$, $\mathbf{X}_2$
- Set connectivity matrix as $\mathbf{C} = \begin{bmatrix} 0 & 0 \\ 1 & 0 \end{bmatrix}$, that is $\mathbf{X}_2$ is response to $\mathbf{X}_1$
- Set $\mathbf{w}_1^{(0)}$ and $\mathbf{w}_2^{(0)}$ to arbitrary vectors $[1, 1, ..., 1]'$ with length p_1 and p_2, respectively
- Define convergence criterion $CRT = 1$ and a small positive tolerance $\gamma = 10^{-6}$

(ii) *Iterative regression steps*

While $CRT \geq \gamma$

a. *Estimate initial LVs*

$\boldsymbol{\zeta}_1 \propto \mathbf{X}_1\mathbf{w}_1^{(0)}$; where $\propto$ indicates that $\boldsymbol{\zeta}_1$ is normalized to unit variance

$\boldsymbol{\zeta}_2 \propto \mathbf{X}_2\mathbf{w}_2^{(0)}$

b. *Model the relationship between data sources*
Let $\mathbf{Z}$ be the matrix of the column-bind LVs, i.e. $\mathbf{Z} = [\boldsymbol{\zeta}_1, \boldsymbol{\zeta}_2]$

(i) Let vector $\mathbf{c}_q$ be the q-th row vector of $\mathbf{C}$ that denotes the explanatory data sources for data source q, i.e. $\mathbf{c}_1 = [0,0], \mathbf{c}_2 = [1,0]$

If $\sum_{i=1}^{2} c_{qi} > 0$, i.e. if data source q has any explanatories:
$\theta_{\mathbf{c}_q,q} = [\mathbf{Z}'_{\mathbf{c}_q}\mathbf{Z}_{\mathbf{c}_q}]^{-1}\mathbf{Z}'_{\mathbf{c}_q}\boldsymbol{\zeta}_q$,
where $\mathbf{Z}_{\mathbf{c}_j}$ is the matrix of column-bind explanatory LVs of data source q, i.e. $||\mathbf{c}_1||^2 = 0$, which implies $\mathbf{X}_1$ doesn't have any explanatory data sources, for $\mathbf{c}_2$, we calculate

$$\theta_{\mathbf{c}_2 q} = [\mathbf{Z}'_{\mathbf{c}_2}\mathbf{Z}_{\mathbf{c}_2}]^{-1}\mathbf{Z}'_{\mathbf{c}_2}\boldsymbol{\zeta}_q$$
$$\theta_{12} = [\boldsymbol{\zeta}'_1\boldsymbol{\zeta}_1]^{-1}\boldsymbol{\zeta}'_1\boldsymbol{\zeta}_2$$

(ii) Let vector $\mathbf{c}_{q'}$ be the k-th column vector of $\mathbf{C}$ that denotes the response data sources for data source q', i.e. $\mathbf{c}_1 = [0,1]', \mathbf{c}_2 = [0,0]'$

If $\sum_{i=1}^{2} c_{iq'} > 0$, i.e. if data source q' has any responses:
$\theta_{\mathbf{c}_{q'},q'} = cor(\boldsymbol{\zeta}_{q'}, \boldsymbol{\zeta}_{\mathbf{c}_{q'}})$,
for $\mathbf{c}_1$ we calculate

$$\theta_{\mathbf{c}_1 1} = cor(\boldsymbol{\zeta}_1, \boldsymbol{\zeta}_{\mathbf{c}_2})$$
$$\theta_{21} = cor(\boldsymbol{\zeta}_1, \boldsymbol{\zeta}_2)$$

After the last two steps, the entries of $\boldsymbol{\Theta}$ will look as follows;

$$\boldsymbol{\Theta} = \begin{bmatrix} 0 & [\boldsymbol{\zeta}'_1\boldsymbol{\zeta}_1]^{-1}\boldsymbol{\zeta}'_1\boldsymbol{\zeta}_2 \\ cor(\boldsymbol{\zeta}_1, \boldsymbol{\zeta}_2) & 0 \end{bmatrix}$$

c. *Re-estimate the the LVs*
$[\tilde{\boldsymbol{\zeta}}_1, \tilde{\boldsymbol{\zeta}}_2] = [\boldsymbol{\zeta}_1, \boldsymbol{\zeta}_2]\boldsymbol{\Theta}$

d. *Estimate the new $\mathbf{w}_q^{(1)}$ weights*
$\mathbf{w}_1^{(1)} = [\mathbf{X}'_1\mathbf{X}_1]^{-1}\mathbf{X}'_1\tilde{\boldsymbol{\zeta}}_1$
$\mathbf{w}_2^{(1)} = \left[[\tilde{\boldsymbol{\zeta}}'_2\tilde{\boldsymbol{\zeta}}_2]^{-1}\tilde{\boldsymbol{\zeta}}'_2\mathbf{X}_2\right]'$

e. *Evaluate the convergence criteria and discard the old $\mathbf{w}_q^{(0)}$ weights*
$CRT = \sum_{q=1}^{2}(\mathbf{w}_q^{(1)} - \mathbf{w}_q^{(0)})^2$
$\mathbf{w}_q^{(0)} = \mathbf{w}_q^{(1)}$

(iii) *Upon convergence, return* $\mathbf{w}_1^{(0)}$ and $\mathbf{w}_2^{(0)}$

In this iterative regression framework, $\tilde{\boldsymbol{\zeta}}_1$ is regressed multivariably on $\mathbf{X}_1$ to obtain the $\mathbf{w}_1$ weights and $\mathbf{X}_2$ is regressed univariately on $\tilde{\boldsymbol{\zeta}}_2$ to obtain the $\mathbf{w}_2$ weights. The algorithm stops when weights converge (i.e. within a small predefined

tolerance which is denoted by γ). It can be shown that this algorithm leads to the characteristic eigenvalue equation of RDA as follows [2]

$$\begin{aligned}\mathbf{w}_2^{(n)} &= [[\tilde{\boldsymbol{\zeta}}_2'\tilde{\boldsymbol{\zeta}}_2]^{-1}\tilde{\boldsymbol{\zeta}}_2'\mathbf{X}_2]' \\ &\propto [\tilde{\boldsymbol{\zeta}}_2'\mathbf{X}_2]',\end{aligned} \tag{1}$$

where the symbol $\propto$ indicates that the left side is proportional to a scaled right side (i.e. $[\tilde{\boldsymbol{\zeta}}_2'\tilde{\boldsymbol{\zeta}}_2]^{-1}$ is a scalar) and superscript n denotes the sequence number of the iteration. Note that after Step (2-b-ii) in the algorithm above, $\tilde{\boldsymbol{\zeta}}_2$ becomes a scaled version of $\boldsymbol{\zeta}_1$. This can be derived from Step (2-c), i.e.

$$\begin{aligned}[\tilde{\boldsymbol{\zeta}}_1, \tilde{\boldsymbol{\zeta}}_2] &= [\boldsymbol{\zeta}_1, \boldsymbol{\zeta}_2]\boldsymbol{\Theta} \\ &= [\boldsymbol{\zeta}_1, \boldsymbol{\zeta}_2]\begin{bmatrix} 0 & [\boldsymbol{\zeta}_1'\boldsymbol{\zeta}_1]^{-1}\boldsymbol{\zeta}_1'\boldsymbol{\zeta}_2 \\ cor(\boldsymbol{\zeta}_1, \boldsymbol{\zeta}_2) & 0 \end{bmatrix} \\ &= \Big[[\boldsymbol{\zeta}_2 \times cor(\boldsymbol{\zeta}_1, \boldsymbol{\zeta}_2)], [\boldsymbol{\zeta}_1 \times [\boldsymbol{\zeta}_1'\boldsymbol{\zeta}_1]^{-1}\boldsymbol{\zeta}_1'\boldsymbol{\zeta}_2]\Big],\end{aligned} \tag{2}$$

where $cor(\boldsymbol{\zeta}_1, \boldsymbol{\zeta}_2)$ and $[\boldsymbol{\zeta}_1'\boldsymbol{\zeta}_1]^{-1}\boldsymbol{\zeta}_1'\boldsymbol{\zeta}_2$ are scalars. Then we can rewrite Eq (1) as

$$\begin{aligned}\mathbf{w}_2^{(n)} &\propto \big(\tilde{\boldsymbol{\zeta}}_2'\mathbf{X}_2\big)' \\ &\propto \big((\boldsymbol{\zeta}_1 \times [\boldsymbol{\zeta}_1'\boldsymbol{\zeta}_1]^{-1}\boldsymbol{\zeta}_1'\boldsymbol{\zeta}_2)'\mathbf{X}_2\big)' \\ &\propto \big(\boldsymbol{\zeta}_1'\mathbf{X}_2\big)' \\ &\propto \big(\mathbf{w}_1^{(n-1)'}\mathbf{X}_1'\mathbf{X}_2\big)' \\ &\propto \big(\tilde{\boldsymbol{\zeta}}_1'\mathbf{X}_1[\mathbf{X}_1'\mathbf{X}_1]^{-1}\mathbf{X}_1'\mathbf{X}_2\big)',\end{aligned} \tag{3}$$

note here that $\tilde{\boldsymbol{\zeta}}_1$ is a scaled version of $\boldsymbol{\zeta}_2$ (from Eq (2)). Therefore

$$\begin{aligned}\mathbf{w}_2^{(n)} &\propto \big(\tilde{\boldsymbol{\zeta}}_1'\mathbf{X}_1[\mathbf{X}_1'\mathbf{X}_1]^{-1}\mathbf{X}_1'\mathbf{X}_2\big)' \\ &\propto \big(\boldsymbol{\zeta}_2'\mathbf{X}_1[\mathbf{X}_1'\mathbf{X}_1]^{-1}\mathbf{X}_1'\mathbf{X}_2\big)' \\ &\propto \big(\mathbf{w}_2^{(n-1)'}\mathbf{X}_2'\mathbf{X}_1[\mathbf{X}_1'\mathbf{X}_1]^{-1}\mathbf{X}_1'\mathbf{X}_2\big)'.\end{aligned} \tag{4}$$

Transposing the right side leads to

$$\mathbf{w}_2^{(n)} \propto \mathbf{X}_2'\mathbf{X}_1[\mathbf{X}_1'\mathbf{X}_1]^{-1}\mathbf{X}_1'\mathbf{X}_2\mathbf{w}_2^{(n-1)}, \tag{5}$$

and, upon convergence of the algorithm results

$$\mathbf{w}_2 \propto \mathbf{X}_2'\mathbf{X}_1[\mathbf{X}_1'\mathbf{X}_1]^{-1}\mathbf{X}_1'\mathbf{X}_2\mathbf{w}_2. \tag{6}$$

The last equation corresponds with the characteristic eigenvalue equation for RDA [3, 4]. Denote $\mathbf{X}_1$ with $\mathbf{X}$ and $\mathbf{X}_2$ with $\mathbf{Y}$ and rearrange Eq (6) as

$$\begin{aligned} \mathbf{Y}^{'}\mathbf{X}[\mathbf{X'X}]^{-1}\mathbf{X}^{'}\mathbf{Y}\mathbf{w}_2 &= \lambda\mathbf{w}_2 \\ (\mathbf{Y}^{'}\mathbf{X}[\mathbf{X}^{'}\mathbf{X}]^{-1}\mathbf{X}^{'}\mathbf{Y}-\lambda\mathbf{I})\mathbf{w}_2 &= 0 \\ (S_{\mathbf{YX}}S_{\mathbf{X}'\mathbf{X}}^{-1}S_{\mathbf{YX}}^{'} - \lambda\mathbf{I})\mathbf{w}_2 &= 0, \end{aligned} \tag{7}$$

where $S_{\mathbf{YX}}$ is the covariance matrix of $\mathbf{Y}$ and $\mathbf{X}$ and $S_{\mathbf{XX}}$ is the variance matrix of $\mathbf{X}$, and $\mathbf{w_2}$ and λ are the eigenvector and eigenvalue of the characteristic RDA equation [3].

Symmetric relationship of two data sources: CCA
In a symmetric relationship, connectivity matrix $\mathbf{C}$ in Section Step (1-b) becomes

$$\mathbf{C} = \begin{bmatrix} 0 & 1 \\ 1 & 0 \end{bmatrix},$$

that is $\mathbf{X}_2$ is response to $\mathbf{X}_1$ and $\mathbf{X}_1$ is response to $\mathbf{X}_2$. $\mathbf{\Theta}$ in Step (2-b) becomes

$$\mathbf{\Theta} = \begin{bmatrix} 0 & cor(\boldsymbol{\zeta}_1, \boldsymbol{\zeta}_2) \\ cor(\boldsymbol{\zeta}_1, \boldsymbol{\zeta}_2) & 0 \end{bmatrix}$$

and $\mathbf{w}_2$ in Step (2-d) is calculated as

$$\mathbf{w}_2^{(1)} = \left([\mathbf{X}_2^{'}\mathbf{X}_2]^{-1}\mathbf{X}_2^{'}\tilde{\boldsymbol{\zeta}}_2\right)^{'}$$

With the new connectivity matrix $\mathbf{C}$ encoding the symmetric relationship type, the algorithm leads to the characteristic eigenvalue equation of CCA as follows [2]:

$$\mathbf{w}_2^{(n)} = \left([\mathbf{X}_2^{'}\mathbf{X}_2]^{-1}\mathbf{X}_2^{'}\tilde{\boldsymbol{\zeta}}_2\right)^{'} \tag{8}$$

where superscript n denotes the sequence number of the iteration. Note that after Step (2-b-ii) $\tilde{\boldsymbol{\zeta}}_2$ becomes a scaled version of $\boldsymbol{\zeta}_1$. This can be derived from Step (2-c), i.e.

$$\begin{aligned} [\tilde{\boldsymbol{\zeta}}_1, \tilde{\boldsymbol{\zeta}}_2] &= [\boldsymbol{\zeta}_1, \boldsymbol{\zeta}_2]\mathbf{\Theta} \\ &= [\boldsymbol{\zeta}_1, \boldsymbol{\zeta}_2] \begin{bmatrix} 0 & cor(\boldsymbol{\zeta}_1, \boldsymbol{\zeta}_2) \\ cor(\boldsymbol{\zeta}_1, \boldsymbol{\zeta}_2) & 0 \end{bmatrix} \\ &= \left[\left(\boldsymbol{\zeta}_2 \times cor(\boldsymbol{\zeta}_1, \boldsymbol{\zeta}_2)\right), \left(\boldsymbol{\zeta}_1 \times cor(\boldsymbol{\zeta}_1, \boldsymbol{\zeta}_2)\right)\right], \end{aligned} \tag{9}$$

where $cor(\boldsymbol{\zeta}_1, \boldsymbol{\zeta}_2)$ is a scalar. Then we can rewrite Eq (8) as

$$
\begin{aligned}
\mathbf{w}_2^{(n)} &= \left([\mathbf{X}_2^{'}\mathbf{X}_2]^{-1}\mathbf{X}_2^{'}\tilde{\boldsymbol{\zeta}}_2\right)^{'} \\
&\propto \left([\mathbf{X}_2^{'}\mathbf{X}_2]^{-1}\mathbf{X}_2^{'}\boldsymbol{\zeta}_1\right)^{'} \\
&\propto \left([\mathbf{X}_2^{'}\mathbf{X}_2]^{-1}\mathbf{X}_2^{'}\mathbf{X}_1\mathbf{w}_1^{(n-1)}\right)^{'} \\
&\propto \left([\mathbf{X}_2^{'}\mathbf{X}_2]^{-1}\mathbf{X}_2^{'}\mathbf{X}_1[\mathbf{X}_1^{'}\mathbf{X}_1]^{-1}\mathbf{X}_1^{'}\tilde{\boldsymbol{\zeta}}_1\right)^{'},
\end{aligned}
\tag{10}
$$

where the symbol $\propto$ indicates that the left side is proportional to the scaled right side. Note that $\tilde{\boldsymbol{\zeta}}_1$ is a scaled version of $\boldsymbol{\zeta}_2$ (from Eq 9). Therefore

$$
\begin{aligned}
\mathbf{w}_2^{(n)} &\propto \left([\mathbf{X}_2^{'}\mathbf{X}_2]^{-1}\mathbf{X}_2^{'}\mathbf{X}_1[\mathbf{X}_1^{'}\mathbf{X}_1]^{-1}\mathbf{X}_1^{'}\tilde{\boldsymbol{\zeta}}_1\right)^{'} \\
&\propto \left([\mathbf{X}_2^{'}\mathbf{X}_2]^{-1}\mathbf{X}_2^{'}\mathbf{X}_1[\mathbf{X}_1^{'}\mathbf{X}_1]^{-1}\mathbf{X}_1^{'}\boldsymbol{\zeta}_2\right)^{'} \\
&\propto \left([\mathbf{X}_2^{'}\mathbf{X}_2]^{-1}\mathbf{X}_2^{'}\mathbf{X}_1[\mathbf{X}_1^{'}\mathbf{X}_1]^{-1}\mathbf{X}_1^{'}\mathbf{X}_2\mathbf{w}_2^{(n-1)}\right)^{'}.
\end{aligned}
\tag{11}
$$

Transposing the right side leads to

$$
\mathbf{w}_2^{(n)} \propto \mathbf{w}_2^{(n-1)'}\mathbf{X_2}^{'}\mathbf{X}_1[\mathbf{X}_1^{'}\mathbf{X}_1]^{-1}\mathbf{X}_1^{'}\mathbf{X}_2[\mathbf{X}_2^{'}\mathbf{X}_2]^{-1},
\tag{12}
$$

and, upon convergence of the algorithm results

$$
\mathbf{w}_2 \propto \mathbf{w}_2^{'}\mathbf{X_2}^{'}\mathbf{X}_1[\mathbf{X}_1^{'}\mathbf{X}_1]^{-1}\mathbf{X}_1^{'}\mathbf{X}_2[\mathbf{X}_2^{'}\mathbf{X}_2]^{-1}
\tag{13}
$$

Eq (13) corresponds with the characteristic eigenvalue equation for CCA [3, 5]. Denote $\mathbf{X}_1$ with $\mathbf{X}$ and $\mathbf{X}_2$ with $\mathbf{Y}$ and rearrange Eq 13 as

$$
\begin{aligned}
\mathbf{w}_2^{'}\mathbf{Y}^{'}\mathbf{X}[\mathbf{X}^{'}\mathbf{X}]^{-1}\mathbf{X}^{'}\mathbf{Y}[\mathbf{Y}^{'}\mathbf{Y}]^{-1} &= \lambda\mathbf{w}_2 \\
(\mathbf{Y}^{'}\mathbf{X}[\mathbf{X}^{'}\mathbf{X}]^{-1}\mathbf{X}^{'}\mathbf{Y}[\mathbf{Y}^{'}\mathbf{Y}]^{-1} - \lambda\mathbf{I})\mathbf{w}_2 &= 0 \\
(S_{\mathbf{YX}}S_{\mathbf{X}'\mathbf{X}}^{-1}S_{\mathbf{YX}}^{'}S_{\mathbf{Y}'\mathbf{Y}}^{-1} - \lambda\mathbf{I})\mathbf{w}_2 &= 0,
\end{aligned}
\tag{14}
$$

where $S_{\mathbf{YX}}$ is the covariance matrix of $\mathbf{Y}$ and $\mathbf{X}$, $S_{\mathbf{XX}}$ is the variance matrix of $\mathbf{X}$, $S_{\mathbf{YY}}$ is the variance matrix of $\mathbf{Y}$ and $\mathbf{w_2}$ and λ are the eigenvector and eigenvalue of the characteristic CCA equation [3].

Author details

References

1. Vinzi, V.E., Russolillo, G.: Partial least squares algorithms and methods. Wiley Interdisciplinary Reviews: Computational Statistics **5**(1), 1–19 (2013). doi:10.1002/wics.1239
2. Höskuldsson, A.: PLS regression. Journal of Chemometrics **2**(August 1987), 581–591 (1988). doi:10.1002/cem.1180020306
3. Legendre, P., Legendre, L.: Numerical Ecology, Volume 24, (Developments in Environmental Modelling). Elsevier Science Amsterdam, The Netherlands (2012)
4. van den Wollenberg, A.L.: Redundancy analysis an alternative for canonical correlation analysis. Psychometrika **42**(2), 207–219 (1977). doi:10.1007/BF02294050
5. Hotelling, H.: Relations Between Two Sets of Variates. Biometrika **28**(3/4), 321 (1936). doi:10.2307/2333955
